# Therapeutic potential of apoptotic vesicles in modulating inflammation, immune responses, and tissue regeneration

**DOI:** 10.1186/s12951-025-03278-1

**Published:** 2025-04-01

**Authors:** Mohammad Amin Khalilzad, Javad Mohammadi, Soumayeh Amirsaadat, Sajad Najafi, Sona Zare, Mohammad Ali Nilforoushzadeh, Mitra Khalilzad, Mohammad Amir Amirkhani, Aysan Peyrovan, Seyedeh Fatemeh Sadati Khalili, Atefeh Farahani, Solmaz Zare

**Affiliations:** 1 Department of Life Sciences Engineering, Faculty of New Sciences and Technologies, University of Tehran, Tehran, 143951561 Iran; 2 Skin and Stem Cell Research Center, Tehran University of Medical Sciences, Tehran, Iran; 3 School of Medicine, Shahid Beheshti University of Medical Sciences, Tehran, Iran; 4 Stem Cell Research Center, Tabriz University of Medical Sciences, Tabriz, Iran; 5 Department of Medical Biotechnology, School of Advanced Technologies in Medicine, Shahid Beheshti University of Medical Sciences, Tehran, Iran; 6 Biotechnology and Medicinal Plants Research Center, Ilam University of Medical Sciences, Ilam, Iran; 7 Laserin Medical Sciences, Shahid Beheshti University of Medical Sciences, Tehran, Iran; 8 Stem Cell and Regenerative Medicine Institute, Sharif University of Technology, Tehran, Iran; 9 Department of Mechanical Engineering, Sharif University of Technology, Tehran, Iran; 10 Skin Repair Research Center, Jordan Dermatology and Hair Transplantation Center, Tehran, Iran; 11 Brain Mapping Research Center, Shahid Beheshti University of Medical Sciences, Tehran, Iran; 12 Department of Pharmaceutics, Faculty of Pharmacy, Tehran University of Medical Sciences, Tehran, Iran; 13 Nanotechnology Research Centre, Faculty of Pharmacy, Tehran University of Medical Sciences, Tehran, Iran

**Keywords:** Apoptotic vesicles, Inflammation, Cancer, Ischemic disease, Regeneration

## Abstract

The process of apoptosis plays a crucial role in tissue homeostasis, immune system regulation, and organ formation. Apoptotic vesicles (ApoEVs) are involved in efferocytosis, the process by which phagocytes ingest dead cells. ApoEVs also have potential therapeutic applications in cancer treatment, ischemic diseases, and their anti-inflammatory properties make them incredibly versatile for medical applications. These vesicles can induce apoptosis in cancer cells, provide tumor antigens for cancer vaccines, and even serve as effective drug delivery systems. Moreover, they can target hypoxic cells, inhibit inflammatory cell death pathways, and promote tissue regeneration. Also, their potential in addressing inflammatory disorders such as gastrointestinal ailments, osteoarthritis, and diabetes is promising. Additionally, ApoEVs can polarize anti-inflammatory immune cells and suppress inflammatory immune responses which make them a viable option for addressing the unmet need for novel anti-inflammatory medications. Despite a wealth of reviews examining the applications of ApoEVs, very few have thoroughly investigated the mechanisms underlying their anti-inflammatory effects. This distinctive approach positions the current review as timely and immensely relevant, illuminating the intriguing ways these entities function beyond their established advantages.

**Graphical Abstract:**

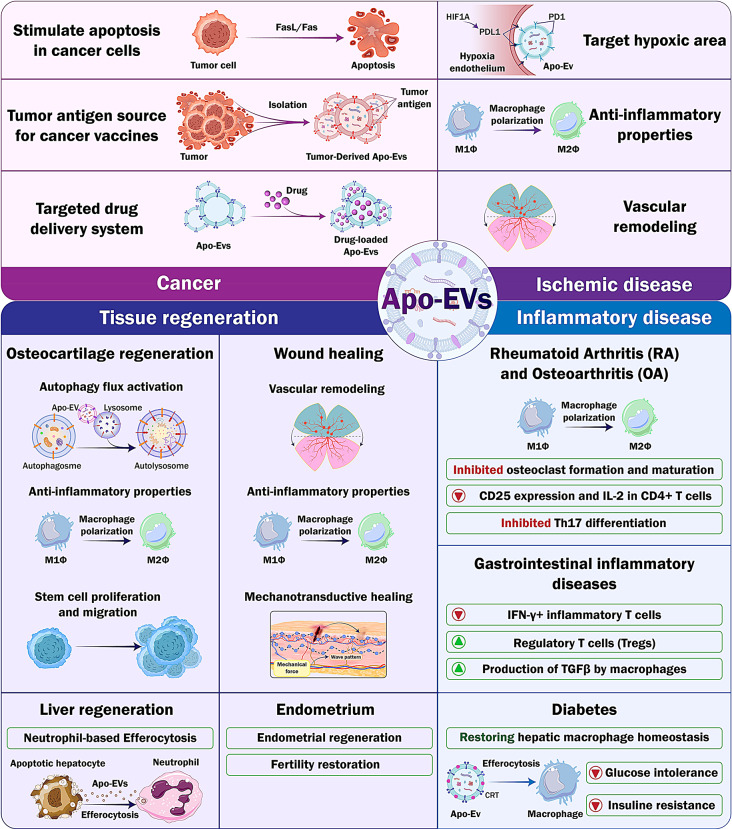

## Introduction

Apoptosis is an essential mechanism in the genesis of organs, tissue homeostasis regulation, and immune system maintenance [1]. Involving the processes of cytoplasmic and nuclear condensation, efferocytosis is the most effective mechanism for phagocytes to ingest deceased cells or their pieces [2]. Both professional and non-professional phagocytes play an essential role in the regulation of the immune system by generating cytokines that facilitate the cooperation of other phagocytes in the process of efferocytosis [3].

Extracellular vesicles (EVs) are lipid bilayer-bound vesicles that stimulate cell signaling, in addition to regulating immune response and tissue homeostasis [4]. These may be classified into exosomes, microvesicles (MVs), and apoptotic vesicles (ApoEVs). Exosomes have a lower immunogenicity, homing efficiency, and extended retention durations compared to other EVs. MVs play a vital role in intercellular communication, immunological control, and signal transmission [5]. ApoEVs are affiliated with the immune system and are actively recruited and ingested by immune cells, particularly macrophages [6]. They play a crucial role in maintaining internal balance, transmitting signals, and stimulating tissue growth. ApoEVs are used for direct therapeutic purposes, as carriers, for vaccinations, and to facilitate diagnostic procedures [7]. In this review, we discuss the use of their anti- inflammatory characteristics in various health conditions.

Defects in apoptosis are closely associated with cancer, making the targeting of apoptosis pathways a cornerstone of cancer treatment. However, the complexity of apoptosis pathways presents significant challenges, as various therapeutic mechanisms can obstruct these treatments. We can effectively address resistance mechanisms by strategically combining targeted agents with innovative approaches. This makes the modulation of apoptosis pathways an incredibly promising avenue for advancing oncology therapies [8].

The inherent characteristics of ApoEVs make them very promising for combating cancer. Initially, they can trigger apoptosis, a programmed cell death process in cancer cells, potentially suppressing tumor growth [9]. Furthermore, they serve as a valuable source of tumor antigens that may be used to develop mature dendritic cells (DCs) for cancer vaccines [10]. This development would, therefore, enhance the presentation of antigens to T cells, thereby promoting their activation. The third use of ApoEVs in anti-cancer treatments is as a drug delivery system, capable of being loaded with various anti-cancer medications and explicitly administered to tumor tissue [11].

ApoEVs offer a promising potential for the treatment of ischemia. They may directly target hypoxia by interacting with markers on hypoxic endothelial cells (ECs). The accurate identification of these markers by therapeutic agents is crucial for developing more precise treatments [12]. Moreover, they possess intrinsic characteristics that support anti-ischemic treatments. One such characteristic is their capacity to impede pathways involved in inflammatory cell death. This inhibition can effectively reduce inflammation in ischemic conditions, which is highly significant in several ischemic diseases like myocardial infarction (MI) [9].

**Fig. 1:**
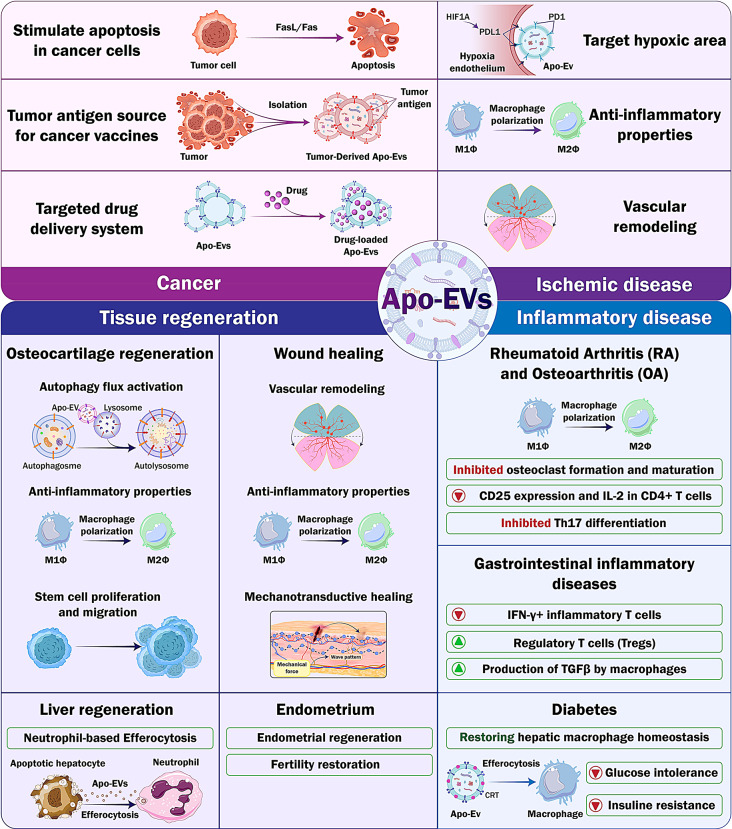
A schematic illustration of therapeutic applications of apoptotic vesicles. In cancer treatment, ApoEVs can work through three main mechanisms: stimulating apoptosis in cancer cells, serving as a source of tumor antigens for vaccines, and acting as a drug delivery system. For ischemic diseases, they can target hypoxic areas, promote M2- macrophage polarization, and facilitate vascular remodeling. ApoEVs also aid in tissue regeneration across various areas, including osteocartilage, wound healing, liver, and endometrium. They achieve this through mechanisms such as activating autophagy flux, promoting stem cell proliferation, and enhancing mechanotransductive healing. Moreover, ApoEVs can help manage various inflammatory diseases, including rheumatoid arthritis, osteoarthritis, gastrointestinal inflammation, and diabetes, by inducing anti-inflammatory macrophage polarization, increasing regulatory T cells, inhibiting inflammatory T cells, and restoring hepatic macrophage homeostasis

ApoEVs possess distinct characteristics that make them a beneficial resource for regenerative applications. Demonstrating the significance of inflammation management in tissue regeneration, it is evident that apoptosis and its related secretions may effectively suppress inflammation. Furthermore, it is well-established that autophagy plays a crucial role in tissue regeneration and rejuvenation. Most recent discoveries indicate that ApoEVs can initiate this process [13]. Furthermore, the ApoEVs and their related products are typically removed by efferocytosis [14]. Neutrophils, an essential type of immune cells, play an important role in the healing processes. Upon encountering and engulfing ApoEVs—cells that have undergone a programmed form of cell death—they initiate a remarkable response. Following this efferocytosis, neutrophils release regenerative cytokines, signaling proteins that promote tissue repair and regeneration. This process clears away dying cells and enhances tissue regeneration by stimulating various healing pathways, highlighting the importance of neutrophils in maintaining homeostasis and promoting recovery within the body [15]. Furthermore, these tiny regeneration compartments include anti-inflammatory capabilities that enable them to specifically target several inflammatory disorders like gastrointestinal ailments, osteoarthritis, and diabetes. One well-documented characteristic is their capacity to polarize anti-inflammatory macrophages and suppress inflammatory immune cells [16]. Furthermore, recent research indicates that the inhibitory effects of ApoEVs on osteoclasts may participate in the inhibition of osteoarthritis [17]. Nevertheless, their anti-inflammatory activities are not restricted to this case. ApoEVs may also engage with T helpers as a key directional component in the immune system. It is proposed that they can suppress type 1 and 17 T helpers, pivotal in inflammatory disorders, and activate regulatory T cells, which are anti-inflammatory cells in the immune system [18]. While numerous reviews have explored the applications of ApoEVs, few have taken a deep dive into the mechanisms rather than their anti-inflammatory properties. This unique focus makes the current review both timely and highly relevant, shedding light on the fascinating ways these entities operate beyond their well-known benefits (Fig. 1).

### Apoptosis and apoptotic vesicles

Organism development, tissue homeostasis, and immune system maintenance depend on the precisely controlled cell death process of apoptosis [1]. The cytoplasmic and nuclear condensation processes are two steps in apoptosis [19]. Efferocytosis is how phagocytes consume dead cells or their pieces, effectively cleaning them up. Preserving the morphological and functional integrity of an organism’s tissues depends on efferocytosis throughout its life [2]. Efferocytosis is more efficient in specialized phagocytes, such as macrophages and DCs. Non-professional phagocytes (specific epithelial cells and fibroblasts) take on a key role when professional phagocytes are inadequate or find it challenging to reach dead cells. The regulation of immune system depends on both professional and non-professional phagocytes. Non-professional phagocytes can produce cytokines that help similar population of phagocytes in distributing the composition of efferocytosis. Conversely, professional phagocytes may generate cytokines to activate other immune cells [3].

The significance of cell death, particularly in the context of apoptosis, has increased substantially due to recent findings suggesting that an adult human loses approximately 50 billion cells daily. In this context, a comprehensive understanding of apoptosis as an anti-inflammatory cell death mechanism may be essential for the effective management of inflammation [6]. Both intrinsic and extrinsic pathways can initiate apoptosis depending on the initial stimulation type. The extrinsic pathway begins with external signals, including the binding of death ligands to cell surface receptors. On the other hand, endogenous signals, typically in reaction to cellular stress or damage activate the intrinsic pathway. The control of cell apoptosis depends critically on the caspase family of proteases [20]. There are two primary categories of caspases: inflammatory caspases and apoptotic caspases. Inflammatory caspases play a crucial role in triggering pyroptosis, a form of programmed cell death (PCD) characterized by the release of pro-inflammatory cytokines, which leads to various inflammatory reactions in the body. On the other hand, apoptotic caspases are essential for regulating the process of apoptosis, ensuring that damaged or unnecessary cells are systematically eliminated. The interplay between these two types of caspases is vital for managing immunological responses in humans and animals, highlighting their importance in maintaining health and responding to disease [21]. One of the primary entities involved in this context is ApoEVs, which are classified as a specific category of EVs.

EVs are lipid bilayer-bound vesicles that originate from cells and play a crucial role in the regulation of immune response, facilitation of intercellular communication, maintenance of tissue homeostasis, and promotion of tumor development [4]. They have three potential classifications: exosomes, MVs, and ApoEVs. Exosomes have low immunogenicity, favorable homing, and possess prolonged retention periods [5]. MVs are crucial in intercellular communication, immunological regulation, and signal transduction [22]. ApoEVs are immune system-connected vesicles that are actively drawn and absorbed by immune cells, especially macrophages [23]. They are essential for internal equilibrium, signal transduction, and tissue development stimulation. ApoEVs are used directly for therapeutic targets, as carriers and in vaccine formulation, as well as for diagnostic strategies, among other purposes [7]. Still, the diverse characteristics of the topic present a significant challenge for pragmatic use in a therapeutic environment. Exosomes, MVs, and apoptotic bodies (ApoBDs) are the three main divisions into which ApoEVs [24]. ApoBDs, made during the apoptosis process, are absorbed by macrophages to prevent cellular damage [25]. MVs participate in the regulation of the immune system, sending signals inside cells, and facilitate the intercellular transduction of messages [26]. Conversely, exosomes are produced from certain endosomal bodies. To investigate the interaction of the immune system and ApoEVs, it is necessary to take a look at S1P/S1PR signaling pathways. G-protein-coupled receptors (GPCRs) that facilitate the S1P (sphingosine-1-phosphate) signaling pathway—namely S1PR1 to S1PR5—play a critical role in the immune response by triggering the activation of proinflammatory cytokines. When S1P binds to these receptors, it initiates vital intracellular processes, including the mitogen-activated protein kinase (MAPK) and nuclear factor kappa (NF-κB)-light-chain-enhancer of activated B cells pathways. This activation produces key proinflammatory cytokines, such as tumor necrosis factor alpha (TNF-α) and interleukin (IL)-6, by immune cells like macrophages and lymphocytes. Furthermore, S1P significantly influences immune cell migration: S1PR1 promotes the release of lymphocytes from lymphoid organs, given its strong association with various inflammatory and autoimmune diseases, this signaling pathway presents a compelling therapeutic target for combating excessive inflammation [27]. Damage-associated molecular patterns (DAMPs) are molecules found within ApoEVs that through S1P/S1PR signaling pathways may activate proinflammatory cytokines, such as IL-1β in macrophages [28].

Apoptosis is the process by which the cell membrane contracts and splits, enclosing broken pieces of the nucleus and cytoplasm in EVs bound to the membrane [29]. These were first seen as ApoBDs. ApoEVs, on the other hand, release specific proteins known as exosomal markers, including tumor susceptibility gene 101 and translationally regulated tumor protein (TCTP). ApoEVs also have irregular shapes that match those of typical exosomes in scale. ApoEV production involves numerous components: protein kinases, cell membrane blebbing, and the synthesis of apoptotic MVs or microparticles. The process depends heavily on caspase activity and is somewhat regulated. Certain essential factors for this process include higher amounts of Bcl-2, adenosine diphosphate (ADP)-ribose polymers, functional microtubules, myosin light chain kinase (MLCK), and the fungal metabolite cytochalasin B. This complex structure emphasizes the need for consistent methods for modifying, defining, and investigating ApoEVs to maximize their therapeutic potential. Further investigation are required to achieve a more precise understanding of the specific signaling components and pathways involved in ApoEV functional efferocytosis, as appropriate usage of ApoEVs in therapeutic environments depends on this information [30].

### Apoptotic vesicles in cancer therapy

The potential of ApoEVs in cancer treatment is significant due to their promising characteristics. Their ability to initiate apoptosis, in cancer cells, is their most important feature, as it hinders tumor growth. Moreover, they provide an essential source of tumor antigens, which makes them suitable for cancer vaccines. This helps promote the development of DCs and enhance antigen presentation to T cells, ultimately improving their activation. Additionally, ApoEVs can serve as vehicles for the delivery of specific drugs, particularly allowing for the precise transport of anti-cancer agents to tumor tissues.

### Mechanisms, applications, and immune modulation

The challenge of overcoming the resistance of tumor cells to PCD is a significant obstacle in cancer therapeutics [31]. To address this challenge, Wang et al. demonstrated that ApoEVs can trigger apoptotic pathways and halt the growth of multiple myeloma (MM) cells. It is worth noting that the administration of mesenchymal stem cell (MSC)-derived ApoEVs in murine models has unveiled a significant increase in their lifespan. With a remarkable approach, ApoEVs facilitate a rapid surge in intracellular calcium flux within MM cells, leading to an elevation in cytosolic calcium levels. This intricate process entails a direct interaction between ApoEVs and MM cells, facilitating the transfer of Fas molecules from the cytoplasm to the cell membrane. When Fas ligands bind to ApoEVs and activate the Fas pathway, it creates a chain of apoptotic cascades within MM cells. This highlights the noteworthy pro-apoptotic effects of ApoEVs [9].

There has been a growing focus on immunotherapy and cancer vaccines to enhance the efficacy of cancer therapies. Specifically concerning cancer vaccines, a critical factor is the sourcing of tumor-specific antigens (TSAs), which presents challenges related to their availability and variability. Another advantageous feature of ApoEVs is their ability to serve as antigen reservoirs, effectively instructing the immune system to recognize and engage with particular antigens in cancer cells. The tumor microenvironment (TME) plays a critical role in the diversity of tumors, as tumor cells use this milieu to create immunological tolerance. Immunosuppression mechanisms are important for the formation of cold and hot tumors. The effectiveness of immunotherapy is enhanced by techniques that focus on the TME modification and transformation of tumor characteristics from cold to hot. Immunotherapy is typically ineffective against cold tumors. Targeted medicines and immunotherapy can potentially revolutionize the field of cancer immunotherapy, especially in the context of “hotness and coldness” tumor tactics. Multi-target medication therapy synergistically enhances the immune response against cold tumors [32]. Tumor vaccination potentially contributes to the activation of anti-cancer immune responses. The production of anti-tumor T cell responses depends on the presentation of antigens by DCs [33]. Scientists have examined DC-specific C-type lectin receptors (CLRs) as potential targets for delivering antigens. Targeting the DC-SIGN receptor as a CLR has shown promising outcomes by guiding its cargo into the major histocompatibility complex (MHC)-I and MHC-II pathways, substantially improving CD8^+^ and CD4^+^ T cell responses. Choosing the suitable antigens is crucial to creating successful vaccinations against cancer. Neo-antigens have shown a great promise in the stimulation of strong immune responses against tumors. A tumor vaccine has been developed by altering the glycosylation structure of melanoma-derived ApoEVs for the exclusive targeting of DCs. This vaccination successfully activated CD8^+^ T cells that recognize antigens, showing that it may be used as a potential strategy in cancer immunotherapy [10].

One potential therapeutic strategy in immunotherapy involves using stimulators of interferon genes (STING) agonists, which can elicit robust innate immune responses [34]. Conventional STING agonists, like 2′,3′-cyclic guanosine monophosphate-adenosine monophosphate (cGAMP), have limited efficacy in addressing the recognition of TSAs due to their inadequate cytosolic transport [35]. A novel nano-vaccine platform was employed to address this concern. The platform comprised nanoparticles (NPs), specifically ApoBDs, with STING-activating and Fenton-reactive properties. This innovative methodology was shown to promote the cooperative generation of ApoBDs from tumor cells. Upon the introduction of exogenous cGAMP encapsulated within ApoBDs, antigen-presenting cells (APCs) efficiently internalized the ApoBDs, like Trojan horses. This mechanism significantly enhanced the immune responsiveness of tumor cells, prompting an inflammatory profile. Consequently, the activation of the STING pathway was markedly augmented, resulting in improved presentation of TSAs. This process, in turn, strengthened adaptive immunity by synergistically engaging with innate immune responses [36].

The heterogeneity in the TME is a significant obstacle to effective targeting of tumor cells with pharmaceutical agents. This poses a substantial barrier to many therapeutic strategies, particularly in the context of cancer treatment [37]. ApoEVs facilitate cell communication and improve their ability to engage with particular cells [30]. Additionally, they can alter the immune response to tumors by transmitting biochemical signals to tumor-associated macrophages (TAMs). Secreted by hepatocellular carcinoma (HCC) cells, EVs promote macrophage M2 polarization. As a result, this process supports the development of HCC by causing monocytes to transform into macrophages and modifying the TME [38]. TAMs exhibit a wide range of characteristics. Specific subgroups, known for their pro-inflammatory (M1-like) characteristics, are crucial in the elimination of malignancies. On the other hand, certain types of immune cells with anti-inflammatory properties (M2-like) play a role in supporting tumor growth and helping cancers avoid detection by the immune system. Caspases 1 and 2 are essential in developing the M1 phenotype in macrophages. This trait is often linked to stimulating the inflammasome complex, including caspase-1, NLR family pyrin domain containing 3 (NLRP3), and apoptosis-associated speck-like protein containing a CARD (ASC). M1-like macrophages have elevated generation of reactive oxygen species (ROS) and significantly depend on glycolytic metabolism, while simultaneously exhibiting reduced levels of oxidative phosphorylation (OXPHOS). TAMs have a significant impact on tumor growth and the effectiveness of immunotherapy. They adapt their polarization in reaction to signals from cancer cells and the TME. In a study within a controlled laboratory environment, Oroxylin A (OA) was encapsulated into ApoEVs to enhance its specific delivery to tumor cells for therapeutic applications. The findings unveiled that OA activates many biochemical pathways in HCC cells, resulting in PCD, commonly known as apoptosis. This research emphasizes the influence of OA on the anti-HCC therapies by the means of M1 polarization, indicating its prospective use in therapeutic immunotherapy [11].

Another concern related to the TME, aside from its heterogeneous structure, pertains to solid tumors and the difficulties associated with the deep penetration of therapeutic agents into these formations. This constraint arises due to the absence of a dependable transportation infrastructure capable of extensive penetration of the intended region [39]. A novel vehicle has been developed to treat tumors with remarkable accuracy, using image-guided navigation to improve precision. This vehicle incorporates contemporary photo-thermal-immunotherapy techniques to enhance treatment effectiveness. The ApoEV is a transporter and vehicle for therapeutic chemicals, whereas IR820 offers a mechanism for guiding fluorescence imaging and regulating photo-thermal effects. This vehicle can convey therapeutic chemicals to malignancies, guaranteeing their profound penetration and facilitating photo-thermal treatment. Moreover, it facilitates the development of in situ vaccinations. Furthermore, the hydrogel that includes CD47 antibodies enhances both the innate and adaptive immune responses by aiding in the control of the immunosuppressive surroundings via macrophage polarization. This carrier has shown encouraging outcomes in mice models of breast cancer (4T1), suggesting its potential as a helpful tool in cancer therapy. This strategy offers a promising answer to the problem of reaching deep tissues, thereby allowing for more precise and effective treatment methods in cancer therapy [40]. In this context, drug encapsulation within these carriers is also essential. Research indicates that the role of Ras-related protein 7 (Rab7) in regulating the efficacy of NP encapsulation in apoptotic MSCs is significant. By stimulating Rab7, there is a promising potential to improve the formation of NP-ApoEVs. This information could be valuable for researchers looking to advance the development of drug loading in vesicles with improved efficiency [41].

Matrix metalloproteinase 2 (MMP2) is a member of the zinc-dependent proteases that degrade some specific components of extracellular matrix (ECM). MMP2 plays a critical role in cancer development by facilitating invasion and angiogenesis, the creation of new blood vessels due to its interactions with cancer and ECs. Many types of cancer frequently express MMP2. In the TME, tumor stromal cells and cancer cells generate MMP2 [42]. This discovery holds significant importance for the medical community, as it could serve as a valuable therapeutic tool and a reliable marker for cancer diagnosis and prognosis. Researchers are working on the development of novel drug delivery systems. By inserting phosphatidylserine (PSL) into NPs that mimic apoptotic entities, a study has found a valuable way to replicate ApoEVs. Unbroken cell membranes often include PSL, a negatively charged phospholipid, on their inner surface. During apoptosis, it migrates to the outer membrane [43]. Upon reaching the tumor site, these NPs facilitate the process of phagocytosis by interacting with TAMs. This is incredibly efficient in regions characterized by excessive MMP2 production. Extensive experimentation to indicate the efficiency of the targeting of TAMs through using various biological models, such as cocultured cell spheroids, cell lines, tumor-bearing animals, co-cultured cells and zebra fish, has consistently shown the exceptional ability of NPs to target TAMs [44]. Zebrafish serve as a valuable model for the evaluation of the safety and effectiveness of NPs in medicine. Their transparent embryos allow for real-time observation of the development processes and the effects of NPs on physiological systems, facilitating the evaluation of toxicity. With genetic similarities to humans, zebrafish help researchers study the interactions of NPs at both cellular and molecular levels, including distribution and therapeutic potential. This model is particularly ideal for high-throughput screening, enabling rapid evaluation of nanomaterials in vivo, which is essential for advancing nanotechnology in drug delivery and cancer treatment [45–47].

Immunotherapy research has determined that ApoBDs, MVs, and exosomes have unique therapeutic possibilities and procedures. One of the most well-known uses for exosomes—which potentially convey therapeutic cargo extremely effectively is the transmission of tumor antigens to APCs, which trigger robust immunostimulatory responses. Exosomes can potentially boost treatment effectiveness, yet their surface protein CD47 may inhibit phagocytosis. On the other hand, MVs, which may range in size from 0.1 to 1.0 μm, are expelled from the plasma membrane, and like exosomes, can possess either immunostimulatory or immunosuppressive properties, depending on their biological source. Although MVs derived from MSCs often have immunomodulatory effects, those originating from cancers may boost immune responses by presenting antigens. Due to their interactions with DCs and macrophages, ApoBDs—50–5000 nm diameter, larger than exosomes and MVs—have a distinct anti-inflammatory potential. One advantage of these interactions for anti-inflammatory treatment is that they induce M2-macrophage polarization. Regarding immunotherapy, all three classes of EVs have promising therapeutic applications; however, ApoBDs stand out from the others due to their distinct anti-inflammatory properties, whereas exosomes and MVs have more varied effects [23] Table 1.

**Table 1:** The application of apoevs in cancer therapy

Source	Condition	Cargo	Results	Reference
Mesenchymal stem cell	Multiple myeloma	--	Induction of tumor cell apoptosis	[9]
Glycan modified Melanoma cell	Melanoma	--	CD8^+^ T promotion	[10]
4 T1 tumor cell	4T1-Luc tumors	cGAMP	STING activation Tumor Ag	[36]
Oroxylin A treated HCC cells	Hepatocellular carcinoma	--	-Tumor cell apoptosis -M1 polarization	[11]
Raw267.4 cells	4T1 tumor-bearing mice	-CD 47 -IR820	Deep tumor penetration	[40]
Cancer cell	--	Genipin-crosslinked nano-adjuvants	-An antigen source and a cargo vehicle -Promoting T-cell infiltration	[48]

### Therapeutic potential of apoptotic vesicles in ischemic diseases: targeting inflammation, tissue remodeling, and macrophage polarization

ApoEVs can significantly affect the treatment of ischemic diseases. They have the potential to specifically target hypoxic regions by interacting with particular markers on hypoxic ECs, which is crucial for the accurate identification of therapeutic targets in ischemic diseases. Additionally, their inherent characteristics, such as their ability to hinder inflammatory cell death pathways, show promise for anti-ischemic treatments. This anti-inflammatory potential is significant in ischemic diseases like MI, where controlling inflammation is substantial.

Cardiovascular ischemic disorders, especially MI, have a significant impact on mortality rates worldwide. Despite the considerable advancements in reperfusion therapy, heart failure remains a substantial concern following MI. Therapeutic interventions like coronary artery bypass surgery and thrombolysis can unintentionally worsen the damage to the already weakened heart muscle. Thus, the timely shift from the inflammatory to the reparative phase after the MI is vital in preventing negative heart changes and facilitating cardiac function recovery [49]. In this regard, the process of efferocytosis is crucial for resolving post-MI inflammation [14]. Nevertheless, certain dying cardiomyocytes and myofibroblasts exhibit resistance to apoptosis, resulting in the release of fibrotic and inflammatory mediators. These mediators contribute to the prolongation of immune responses and the occurrence of harmful structural alterations in the heart [50]. To ensure a smooth transition from the inflammatory to the reparative phase, it is crucial to administer drugs that encourage macrophage polarization directly to the affected myocardial tissue. In this context, Lee et al. have created apoptotic nanovesicles (ApoNVs)—nanovesicles that imitate ApoEVs—to tackle myocardial ischemia-reperfusion (IR) injury. These ApoNVs were specifically engineered to interact with macrophages in the ischemic myocardium through integration of dextran and ischemic cardiac homing peptide (CHP), resulting in powerful immunomodulatory effects. When given intravenously, ApoNV had a remarkable ability to target the ischemic myocardium. Macrophages engulfed them once they reach the ischemic myocardium, resulting in a notable decrease in acute inflammation. Using targeted immunomodulatory ApoNVs in this way can potentially treat inflammatory conditions like myocardial ischemia/reperfusion (IR) injury [51]. When considering the findings of this study, it is essential to raise two questions that can spark a widespread discussion about the therapeutic properties of ApoEVs. The first question is how they can accurately target the ischemic area, while the second question is about the mechanism by which ApoEVs contribute to tissue remodeling. To address these questions, we incorporate a study investigating the application of ApoEVs in retinal vascular remodeling. This study suggested that programmed cell death protein 1 (PD1) carried by ApoEVs interacts with programmed death-ligand 1 (PDL1) on hypoxic ECs, thereby regulating angiogenic activation. This mechanism reveals the potential for them to reach hypoxic microenvironment, a hallmark of various pathological conditions such as MI, cancer, and wound healing. This discovery highlights the potential of ApoEVs as a precise vector for targeted therapy. Another significant discovery from this study was analyzing how these ApoEVs can impact tissue remodeling. The study reveals how these vesicles can effectively target cell metabolism by inhibiting glycolysis, leading to vascular remodeling [12]. To have a deeper insight into the various mechanisms participating in the tissue remodeling process, we can explore the study conducted by Yu et al., in which they evaluated the significance of ferroptosis in ischemia-damaged tissues. Ferroptosis is a distinct form of PCD that predominantly impacts iron metabolism. It is characterized by the buildup of lipid peroxides on cell membranes. This process employs the cystine/glutamate antiporter system xc^−^, which is essential for the ferroptosis process [52]. It activates following an injury, causing a simultaneous elevation in glutamate levels and a reduction in cysteine levels. Lack of cysteine makes recombinant glutathione peroxidase 4 (GPX4) less effective and stops the production of glutathione that is an important antioxidant controlled by recombinant thioredoxin reductase 1 (Txnrd1). The primary factors contributing to ferroptosis are the buildup of ROS and the oxidation of cellular membranes. Fe^2+^ and acyl-CoA synthetase long-chain family member 4 (ACSL4) are crucial in facilitating these activities [53]. A strong link has been found between ferroptosis and macrophage polarization. This is because ferroptosis activates DAMPs, a vital part of this link. These signals indicating internal damage are known as DAMPs, with high mobility group box protein 1 (HMGB-1) and 8-hydroxy-2’-deoxyguanosine (8-OHdG) being famous examples. The signals cause M1- macrophages to become more polarized, releasing pro-inflammatory cytokines and raising the ROS level, which improves ferroptosis. The Kelch-like ECH-associated protein 1-Nuclear factor erythroid 2-related factor 2 (KEAP1-Nrf2) axis is critical for reducing oxidative stress, which comes from various internal and external sources [54, 55]. EVs have shown a lot of promise in targeting ferroptosis by precisely changing the Nrf2 protein. This activity raises the expression of the *SLC7A11* gene, which is essential for the synthesis of antioxidants [56]. The first transcriptome analysis suggests that the KEAP1-Nrf2 pathway could aid in regulating ferroptosis and macrophage polarization in ischemia-damaged tissues.

Furthermore, microRNAs play a critical role in the pathways involved in disease development. The study of miR-328-3p shows that ApoBDs significantly affect the process of bone marrow MSCs (BMSCs) proliferation [57]. Yu et al. reported that using ApoBDs generated from fibroblast-like cells in subcutaneous connective tissue (FSCT) significantly improved distal ischemic flap survival. This research demonstrated the substantial impact of these antioxidants on the reduction of oxidative stress and cellular death. Studies have also shown that they may help change macrophages from the M1 phenotype to the M2 phenotype, which suggests that they may be able to improve a coordinated immune response. An evident change occurred in this process, marked by a decrease in the expression of KEAP1 and the translocation of Nrf2 into the nucleus. This impeded the development of ferroptosis in both ECs and macrophages. By precisely targeting of KEAP1 using miR-339-5p, microRNA sequencing has led to considerable therapeutic effects [58].

### Apoptotic vesicles in regenerative medicine: mechanisms and therapeutic applications across different tissues

The unique characteristics of ApoEVs make them valuable for regenerative purposes. For instance, regulation of inflammation is crucial for tissue regeneration, while the apoptotic process and its associated secretions have the potential of effective reduction of inflammation. Substantial evidence supports the idea that autophagy plays a central role in tissue regeneration and rejuvenation. Recent discoveries suggest that ApoEVs can initiate this process. Furthermore, ApoEVs and their related products are typically removed through efferocytosis. Neutrophils, an essential cell type involved in this process, can produce regenerative cytokines following the efferocytosis of ApoEVs, representing another essential regenerative mechanism associated with ApoEVs.

### Osteocartilage regeneration

Reduced bone density, increased adiposity in the marrow, and impaired BMSCs define aging skeletons [59]. During the process of apoptosis, several ApoEVs linked with senescence-related abnormalities are generated. It has been revealed that young MSC-derived ApoEVs may effectively rejuvenate the nuclear abnormalities of elderly BMSCs and activate autophagy to restore their lowered capacities for osteo-/adipogenic lineage differentiation and self-renewal. Apoptotic young MSCs generated and concentrated a high concentration of Rab7 into ApoEVs, which were later repurposed by recipient old MSCs, hence reestablishing autolysosome formation and supporting autophagy flux activation and MSC rejuvenation. In elderly mice, the systematic infusion of ApoEVs generated from young MSCs restored recipient MSC function, lowered adiposity in the marrow and enhanced bone mass. This shows the role of ApoEVs in rejuvenating old MSCs by reestablishing autolysosome production and presents a feasible therapy approach for age-related bone loss [13].

The proliferation, differentiation, and self-renewal capacities of MSCs are greatly affected by the levels of oxygen present. Usually, MSCs are exposed to normoxia, which is oxygen levels of 21%, much higher than the usual oxygen levels [60]. Lower oxygen levels provide a conducive environment for cartilage, improve the regeneration capabilities of MSCs derived from adipose tissue, and accelerate their growth. Furthermore, diminished oxygen levels also play a role in the regulation of stem cell release [61]. Hypoxia-preconditioned MSCs release EVs, which have shown a promising efficacy for the treatment of osteoarthritis, fracture repair, as well as for skin rejuvenation [62]. In this regard, a recent study focused on investigating the potential of ApoEVs produced by adipose-derived MSCs to enhance the repair of cartilage tissue and their therapeutic advantages [63]. One significant drawback of intra-articular injection is the fast biodegradation of ApoEV suspensions inside the synovial cavity of the knee joint. Decellularized ECMs, such as gelatin, and hydrogels, are optimal choices for the delivery of EVs [64]. Initial investigations have shown that scaffolds created by 3D printing using decellularized chondrocyte ECM have an exceptional biocompatibility and porosity. Consequently, these scaffolds provide a conducive setting for BMSCs [65]. Adipose tissue-derived MSCs generate ApoEVs, known as H-ApoEVs, when exposed to hypoxia. These H-ApoEVs have a more significant impact on the cartilage regeneration when compared to EVs produced under normal oxygen circumstances in terms of stem cell proliferation and migration augmentation, as well as immunomodulatory effects on tissue microenvironment by macrophage polarization towards M2 regenerative phenotype. A gelatin matrix that has been altered and merged with a 3D-printed ECM scaffold was used as a carrier to transport H-ApoEVs into the joint cavity. As a consequence, a technique was developed to regenerate cartilage. This new approach confirmed the effectiveness of H-ApoEVs in therapy and greatly enhanced the process of cartilage regeneration [63]. Other investigations have underlined the importance of hypoxia even more since oxygen-starved ApoEVs demonstrate improved pro-angiogenic efficacy via a cascade of events. One such mechanism is miR-210-3p, which increases the repressed Akt expression and thus, promotes the migration of ECs and improves collagen deposition. This impact is of great relevance in tissue remodeling, one of the essential components of wound healing [66].

Recent findings validate the pro-M2 polarization and regenerative effects of ApoEVs. ApoEVs are vital in attracting and activating phagocytic cells, especially macrophages. They are crucial for the maintenance of tissue homeostasis, modulation of the immune response, and inflammation mitigation [67]. In the context of osteonecrosis, bisphosphonate-related osteonecrosis of the jaw (BRONJ), the diminished migratory capacity of macrophages primarily hampers the effectiveness of current treatment approaches. Scientists created hydrogels by blending ApoEVs with catechol-conjugated chitosan and cerium (Ce)-doped mesoporous bioactive glass NPs (Ce-MBGNs) to tackle this issue. The hydrogels, which comprise 1% of the total weight of Ce-MBGNs, are non-biodegradable and non-cytotoxic. The hydrogels attracted inflammatory macrophages and facilitated their M2 polarization by manipulating macrophage polarization via Ce-MBGNs and enhancing macrophage chemotaxis utilizing ApoEVs. Experiments conducted on mice using a Zometa-induced BRONJ model showed that the combination of Ce-MBGNs and ApoEVs not only enhanced M2 polarization but also reduced M1 polarization. Consequently, there was an increase in the formation of new bone, improved healing of the mucosal tissue, and decreased osteonecrosis. The results emphasize the potential therapeutic benefits of Ce-MBGNs and ApoEVs in supporting tissue regeneration and repair in an inflammatory environment that can provide a new therapeutic approach for BRONJ [68].

**Fig. 2:**
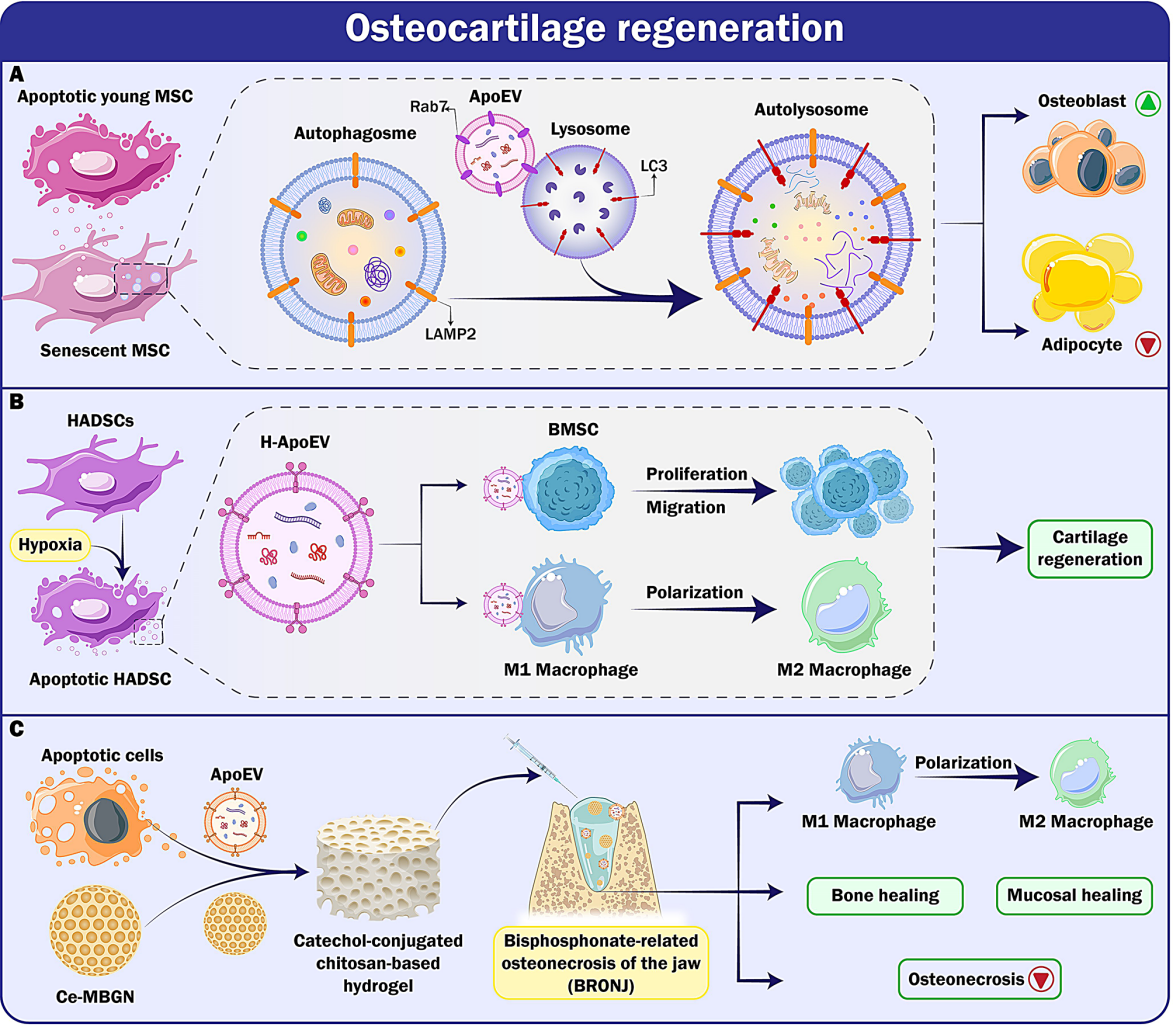
Schematic illustration of the implementation of apoptotic vesicles in osteocartilage regeneration. **A**) Apoptotic vesicles (ApoEVs) from young mesenchymal stem cells (MSCs) enhance chondrocyte function, promote cartilage repair, and increase bone mass by stimulating autophagy. To rejuvenate aged MSCs, the cooperation of LAMP2, LC3, and Rab7 is essential. Rab7 improves autophagic flow and helps form autolysosomes by enriching ApoEVs, while LAMP2 and LC3 promote the fusion of autophagosomes to break down cellular debris. **B**) A cartilage regeneration system is established when HADSCs produce hypoxia apoptotic EVs (H-ApoEVs), which dramatically improve cartilage repair through stem cell proliferation, migration, and M2 polarization. **C**) Ce-MBGN and ApoEV-containing hydrogels promote macrophage polarization, accelerate mucosal healing, promote bone regeneration, and lessen osteonecrosis. LAMP2, Lysosomal-associated membrane protein 2; LC3, Microtubule-associated protein 1 A/1B-light chain 3; H-ApoEVs, hypoxia exposed ApoEVs; HADSCs, human adipose derived stromal cells; Ce-MBGNs, Ce-doped mesoporous bioactive glass NPs; BRONJ, bisphosphonate-related osteonecrosis of the jaw

Another crucial aspect of these vesicles, in addition to the precondition and drug-loading process, is their source of extraction. This might have a significant impact on their ability to regenerate. For example, we have discussed several research studies in this section showing the pro-osteogenic qualities of ApoEVs. However, a recent research contradicts this finding. Macrophage-derived ApoEVs suppressed the bone formation process and boosted adipocyte formation in MSCs in laboratory settings and living organisms. During the mechanism process, ApoEVs were increased explicitly in microRNA-155 (miR-155), and these ApoEVs played a role in the regulation of the development of bone and adipocytes in MSCs by transporting miR-155. In addition, miR-155 regulated the process of bone formation and adipose tissue formation in MSCs grown with ApoEVs produced from macrophages via the SMAD2 signaling pathway [69]. This highlights the importance of the source of vesicles that play a crucial role in therapeutic approaches. However, we hypothesize that using ApoEVs derived from stem cells may have more extensive regenerative effects compared to mature cells. We will discuss this in detail in the subsequent sections of the paper (Fig. 2).

### Wound healing

ECs are critical in wound healing because they significantly contribute to wound closure. Apoptotic ECs create EVs that play crucial roles in the modulation of endothelial gene expression, inflammatory signaling, and cell function. They elicit a physiological state in ECs that promotes their viability and motility, while impeding angiogenesis, suggesting a deterioration in the unique characteristics of ECs [70]. The ApoEVs treatment had contrasting impacts on the cell migration and angiogenesis, indicating the presence of a separate entity that exerted a distinctive and prominent influence on endothelial function. The research demonstrates that ApoEVs induce the dedifferentiation of ECs, resembling the first stage of endothelium-to-mesenchymal transition (endoMT), but does not progress to a mesenchymal state [71]. Migneault and associates have shown that the ApoEVs, produced by apoptotic ECs, may induce functional and phenotypic alterations in the neighboring ECs. The study identified NF-κB binding sites in the promoter region of genes that showed varying expression levels. The activation of NF-κB played a vital role in the functional alterations caused by ApoEVs. The research also found that ApoEVs can stimulate many receptors, including toll-like (TLR) and RIG-I-like receptors, which subsequently initiate the NF-kB pathway. This finding presents new opportunities for the mitigation of endothelial dysfunction and regulation of gene expression in a manner that promotes endothelial dysfunction and mesenchymal transition [72]. Inflammation is the second component of the wound-healing process and plays a significant role in the closure of wounds.

Chronic wounds lead to the accumulation of monocytes and macrophages, which impairs wound healing. The issue is worsened by the ongoing inflammasome activity of wound macrophages, particularly NLRP3. Studies suggest that the activation of the NLRP3 cascade, leading to the death of macrophages by pyroptosis, might significantly contribute to the delayed wound healing seen in type 2 diabetes (T2D) [73]. Various types of cell death are intricately regulated and maintained in a state of equilibrium. Pyroptosis is a specific form of PCD occurs secondary to inflammation and amplifies the inflammatory response. On the other hand, apoptosis is a PCD process associated with anti-inflammatory effects [74]. Evidence indicates that the pyroptosis marker protein gasdermin D (GSDMD) is inactive when apoptotic caspase (caspase-3/7) is activated. Umbilical cord MSCs (UCMSCs) may assist in tissue regeneration and wound healing without invasive surgery. ApoEVs derived from UCMSCs have been indicated to enhance the healing of skin wounds in mice by inhibiting macrophage pyroptosis [75]. As we mentioned, the cell source of ApoEVs plays a crucial role in their therapeutic effects. Recent findings indicate that human embryonic stem cells (ESCs) and induced pluripotent stem cells (iPSCs) generate a greater quantity of ApoBDs compared to human umbilical cord mesenchymal stem cells (hUMSCs). ESC-ApoEVs acquired pluripotent-specific molecules SRY-box 2 (SOX2) from ESCs via a process that relies on caspase 3. In addition, the promotion of mouse skin wound healing may be achieved by introducing SOX2 into skin MSCs, activating the Hippo signaling pathway. These results indicate that ApoEVs can acquire pluripotent molecules from ESCs to activate adult stem cells, therefore highlighting the possibility of using pluripotent stem cells for therapeutic purposes [76].

**Fig. 3:**
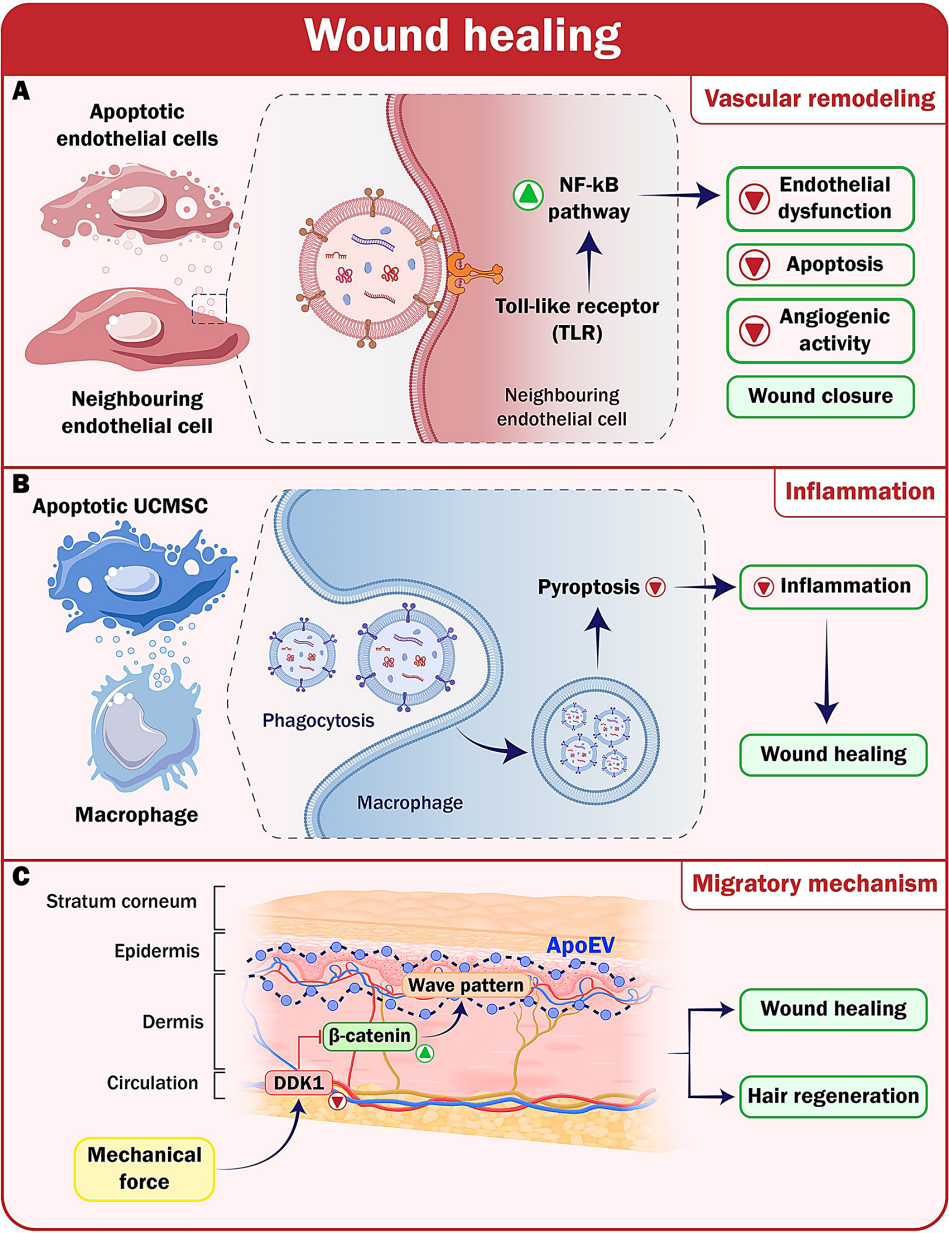
Schematic illustration of the mechanistic implementation of apoptotic vesicles in various aspects of wound healing. **A**) ApoEVs activates NF-κB through TLR, which alters endothelial gene expression. This leads to reduced endothelial markers, enhanced wound closure, and inhibited apoptosis. **B**) ApoEVs from UCMSCs can improve skin wound healing in mice by reducing macrophage pyroptosis, thereby lowering inflammation. **C**) Exogenous apoEVs activate the Wnt/β-catenin pathway during mechanical force by lowering Dickkopf-1 (DKK1), a Wnt signaling inhibitor crucial for wound healing. This mechanical signal increases apoEV migration, enhancing wound healing and promoting hair regeneration

Ma and colleagues conducted a pioneering examination to study the migratory mechanisms of ApoEVs in the circulation and their subsequent mechanism of action. The researchers made an intriguing observation: they found that participating in treadmill exercises significantly enhanced the movement of ApoEVs within the body. Conversely, when the subjects were suspended by their tails, this natural movement was notably impeded, suggesting that the position of the body can influence the circulation and activity of these important biological entities.The production of Dickkopf-related protein 1 (DKK1) in the bloodstream is regulated by mechanical force and is strongly associated with this phenomenon. Exogenous ApoEVs stimulate the activation of the Wnt/β-catenin signaling pathway in MSCs isolated from skin and hair follicles, thereby promoting wound healing and hair development (Fig. 3). This study has revealed that mechanical signaling plays a crucial role in enhancing the wound-healing properties of ApoEVs based on the Wnt/b-catenin pathway. This discovery can revolutionize the wound healing strategies through the integration of mechanotransduction with biochemical cues like ApoEVs [77].

### Other regenerative applications

Researchers have investigated partial hepatectomy (PHx) as a therapeutic approach to induce hepatocellular regeneration; however, we want to interpret this regeneration process through ApoEVs. Efferocytosis triggers an immunological response in phagocytes exhibiting anti-inflammatory and regenerative properties [14]. Recent research has discovered that PHx-induced apoptosis significantly discharges apoptotic cell debris into the bloodstream. Neutrophils were the primary cells that removed ApoEVs under controlled laboratory conditions. These white blood cells developed a pro-regenerative nature as a result of absorbing EVs. According to Brandel et al., the release of ApoEVs has distinct effects on liver regeneration after PHx. The inflammatory and pro-regenerative characteristics of neutrophils stimulate this process. The circulating ApoEVs increased on the first day following the surgical procedure. This rise was associated with elevated levels of apoptosis marker caspase-cleaved cytokeratin-18 (M30), a phenomenon that was only observed in patients who had fully recovered their liver function. Neutrophils participating in efferocytosis produce several growth factors, such as fibroblast growth factor-2 (FGF2) and hepatocyte growth factor (HGF). Therefore, these cells can contribute to a significant increase in liver regeneration [15].

**Fig. 4:**
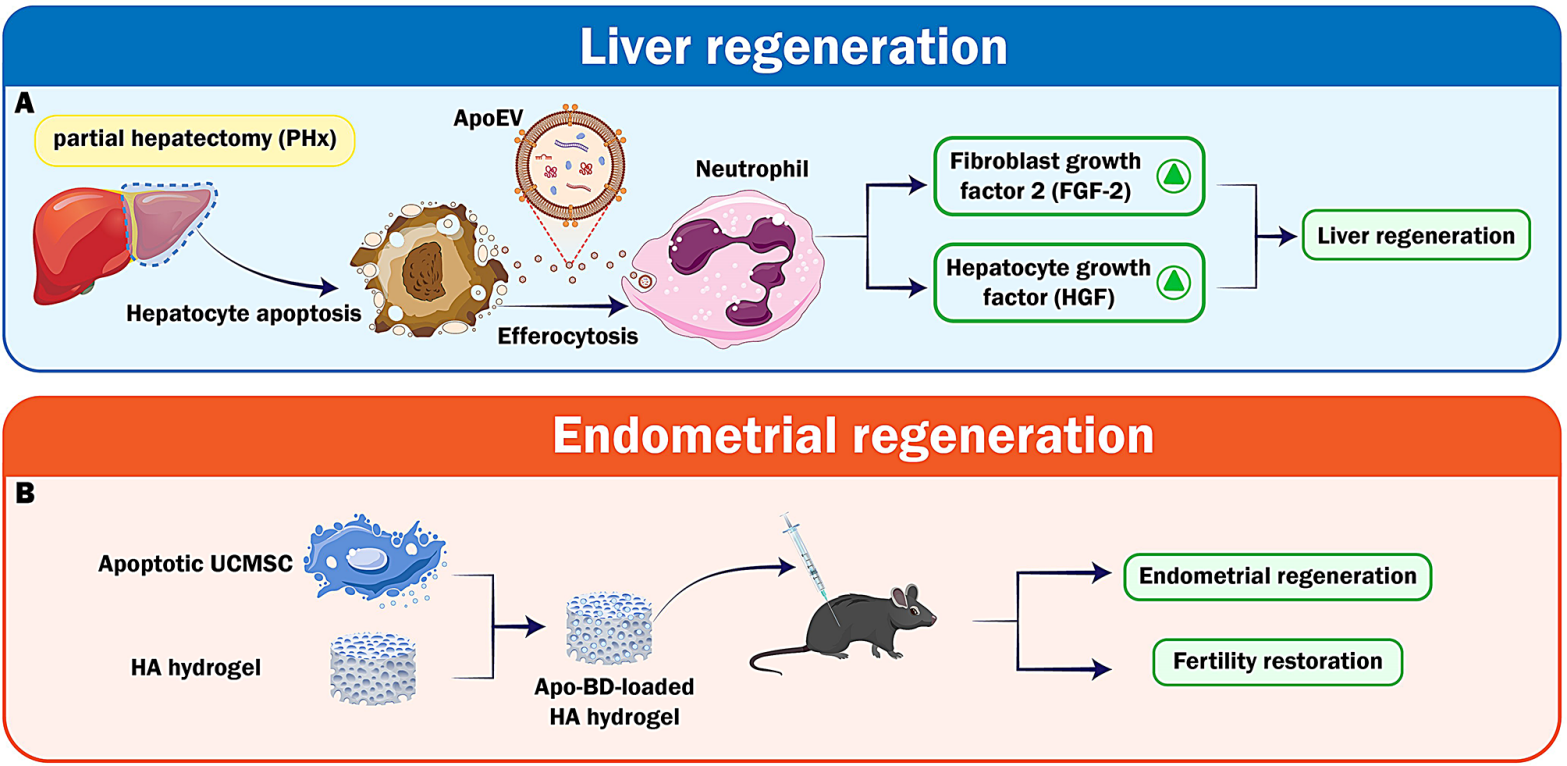
Schematic illustration of the implementation of ApoEVs in liver and endometrial regeneration. **A**) ApoEVs stimulate neutrophil-mediated growth factor release and hepatocyte proliferation, which aid in liver regeneration after Partial hepatectomy. **B**) When endometrial adhesions occur, they also restore endometrial function using ApoBD-loaded hydrogels, which promote collagen remodeling and tissue healing. HA, Hyaluronic Acid

Approximately 5% of women in their reproductive years have recurrent miscarriages, with a majority of them undergoing curettage as a treatment. Infertility is associated with intrauterine adhesions (IUAs) due to severe damage to the basal layer of the endometrium. Traditional remedies for this condition include estrogen therapy, intrauterine devices, and endometrial synechiotomy. Reconstructing a fully functioning endometrium remains a significant challenge, particularly in cases of moderate and severe severity when the likelihood of IUA recurrence is high [78]. A hyaluronic acid (HA) hydrogel has been used as a matrix for ApoBDs, showcasing an excellent functionality and injectability. This study produced a hydrogel containing ApoBDs and HA, which was then tested on mice and rats with acute endometrial damage and IUA. The evaluation focused on regeneration, collagen remodeling, endometrial receptivity, and fertility restoration (Fig. 4). The ApoBD-loaded HA hydrogel was suggested as a promising and effective alternative for endometrial regeneration and a therapeutic option for IUA [79].

### Apoptotic extracellular vesicles: novel therapeutic approaches for autoimmune diseases

The ApoEVs have the capacity to address specific inflammatory conditions, such as gastrointestinal diseases, osteoarthritis, and diabetes, due to their anti-inflammatory properties. Importantly, their proven ability to switch off pro-inflammatory macrophages and activate anti-inflammatory cell phenotypes is a critical feature. Recent research also suggests that they can hinder the activity of osteoclasts, which are vital players in osteoarthritis. Aside from their anti-inflammatory properties, these compartments can also engage with T helper cells, essential in the immune system. It is suggested that ApoEVs can suppress type 1 and 17 T helper cells, which are significant in inflammatory diseases, while also boosting regulatory T cells, known for their anti-inflammatory functions in the immune system.

### Rheumatoid arthritis and osteoarthritis

Rheumatoid arthritis (RA) is a prevalent autoimmune disorder affecting around 5–10% of the global population, and results in lasting joint deformities and disabilities [80]. Currently, the primary method of treatment is the administration of disease-modifying anti-rheumatic drugs (DMARDs) to relieve pain and slow down the advancement of joint degeneration. Glucocorticoids, renowned for their anti-inflammatory properties, are often used in the management of RA. However, the use of these substances has been associated with adverse outcomes, such as an increased susceptibility to infection and the onset of osteoporosis. Recent researches have reported the use of anti-inflammatory NPs; nevertheless, their application is limited [81]. There is a suggestion that ApoEVs might improve the immunological environment and facilitate the repair of bone and cartilage, thus restoring the overall balance of the joint. The design of a study by Li and colleagues was to examine the therapeutic efficacy of ApoEVs derived from macrophages and osteoclasts in treating RA. The in vitro results showed that both ApoEVs sourced from macrophages and osteoclasts induced macrophage polarization towards the anti-inflammatory M2 phenotype, improved chondrocyte function and chondrogenesis, and inhibited osteoclast formation and maturation. Experiments on a mouse model have shown that apoEVs can carry out many functions and collectively impact the joints afflicted by RA. This suggests that ApoEVs have the potential to serve as a promising alternative for RA treatment [17].

As previously mentioned, macrophages play a crucial role in the progression of OA by regulating inflammation and tissue regeneration, preventing pro-inflammatory M1-macrophages, and stimulating anti-inflammatory M2-Macrophages, which can potentially reduce the inflammation associated with OA and stimulate cartilage regeneration. However, it is essential to understand how ApoEVs polarize macrophages to improve the effectiveness of future therapy endeavors. To this end, a study using a mouse model of OA explored how ApoBDs of M2-macrophages (M2-ABs) regulate the balance between M1- and M2-macrophages. The results indicated that M2-ABs may be selectively absorbed by M1-macrophages, leading to a phenotypic reprogramming of M1 to M2 that lasts 24 h. It has surfaced that M2-ABs had a protective effect against chondrocyte mortality in mice, resulting in a decrease in the severity of OA and a reduction in the pro-inflammatory environment caused by M1 activation. The inhibition of miR-21-5p in M1 macrophages successfully decreased the M2-ABs-guided M1-to-M2 reprogramming. According to this research, ApoBDs formed by M2- macrophages may counteract the inflammatory response caused by M1- macrophages by implementing specific microRNAs. These ApoBDs, in turn, can prevent articular cartilage degeneration and improve walking difficulties in animals with OA [16]. The accumulation of apoptotic chondrocytes in OA might disrupt the equilibrium of macrophage homeostasis, leading to an imbalance in the immunosuppressive effects of joints [82]. Researchers have created optically structured NPs decorated with PSL to reprogram macrophages and reduce inflammation. The modulation of M1/M2 polarization has shown that ApoBDs-inspired nanoliposomes containing PSLs might enhance their anti-inflammatory and healing characteristics, thereby promoting the healing of chronic wounds [83]. However, the impact of externally derived nanotherapeutics, inspired by ApoBDs, on chronic inflammatory illnesses with inadequate removal of dying cells, such as OA, remains primarily uncertain. BRD4, a bromodomain-containing protein, acts as an epigenetic regulator of many genes involved in inflammation via super-enhancers and NF-κB signaling [84]. Research has shown that it is highly expressed in OA cartilage, exerting a regulatory impact on the HMGB1 and NF-κB signaling pathways throughout the disease progression. Blocking BRD4 has decreased the degenerative changes similar to OA caused by mechanical stress, lowering the inflammatory response mediated by the triggering receptor expressed on myeloid cells 1 (TREM1) [85]. However, the suppression of BRD4 can potentially trigger senescence in other cell types, including chondrocytes. In a particular study, Xu and colleagues developed JQ1-loaded polymer-stabilized liposomes (JQ1@PSLs) as nano-therapeutics for the targeting of macrophage polarization regulated by BRD4. The study investigated whether JQ1@PSLs, inspired by ApoBDs, can impede the progression of OA in a surgically induced mouse model. Additionally, it examined the role of BRD4 in regulating macrophage polarization during synovitis associated with OA [86].

**Fig. 5:**
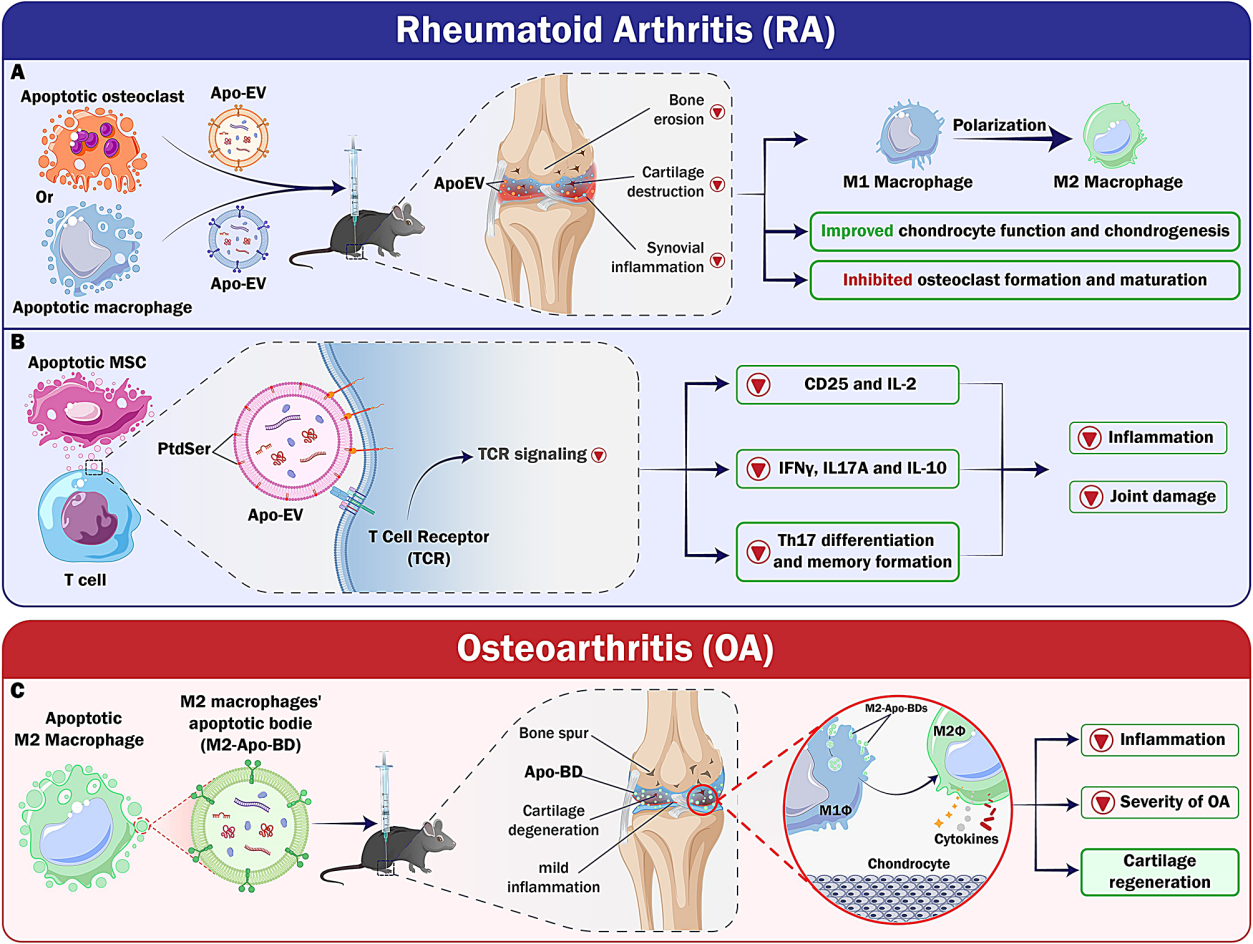
Targeting Rheumatoid Arthritis and osteoarthritis through anti-inflammatory properties of ApoEVs. **A**) APoEVs derived from macrophages and osteoclasts can polarize anti-inflammatory macrophages and inhibit osteoclast formation. **B**) Through direct interaction with CD4 + T cells, ApoEVs suppress the expression of CD25 and the generation of IL-2. Phosphatidylserine exposure on ApoEVs interferes with the transduction of proximal T cell receptor signaling, inhibiting Th17 subsets and associated cytokines, such as IFNγ, IL17A, and IL-10, which controls inflammation in the joints. **C**)M2-ApoBDs targeted by M1- macrophages reprogram M1 to M2 phenotypes, reduce osteoarthritis severity. PtdSer/PS, Phosphatidylserine

Apart from macrophage polarization, recent studies have underlined the immunomodulatory effects of ApoEVs, opening innovative therapeutic opportunities. Several studies have demonstrated that essential immune system components, especially T cells and ApoEVs, interact in multiple ways that regulate immunological responses. ApoEVs treatment in Fas-mutant mice restored ApoEVs levels, addressing aberrant lymphocyte development and insufficiency. This strategy thereby helped lower lupus activity and inflammation. The direct interaction between ApoEVs and CD4^+^ T cells reduced CD25 expression and produced IL-2 in manner dependent on the degree of ApoEVs. This suppression also impacted other subsets of T-helper cells (Th1/2/17) and cytokines like IFNγ, IL17A, and IL-10, maintaining forkhead box P3 (FOXP3) ^+^-regulating T-cells. The functional T cell interaction by exposed phosphatidylserine (PtdSer/PS) on ApoEVs hampered T cell receptor (TCR) signaling. In arthritis models based on animals, the way that ApoEVs repressed Th17 differentiation and memory formation significantly reduced inflammation and joint damage. These findings indicate a previously unknown link between CD4^+^ T cells and ApoEVs generated from MSCs, implying great opportunities for ApoEVs in the treatment of autoimmune disorders [18]. This study emphasizes their complex and flexible character in modifying immune responses and provides a new understanding of the processes by which ApoEVs works. Through a direct interaction with CD4^+^ T cells, ApoEVs can reduce the generation of pro-inflammatory cytokines and stimulate the activation of regulatory pathways crucial for the preservation of immunological balance. Given the critical role Th17 cells play in autoimmune disease, the ability of ApoEVs to target the development and memory formation of Th17 cells has tremendous relevance (Fig. 5).

### Gastrointestinal inflammatory disease

The polarization of macrophages plays a critical role in inflammatory conditions affecting the gastrointestinal tract. This polarization influences the balance between pro-inflammatory and anti-inflammatory responses. In the context of inflammatory bowel disease (IBD), an increase in M1- macrophages may exacerbate inflammation and damage the intestinal lining, whereas enhanced M2 polarization has been associated with an improved healing and restoration of gut homeostasis. Research indicates that the therapeutic strategies aimed at shifting macrophage polarization from M1 to M2 may be effective in treating IBD by enhancing anti-inflammatory responses and facilitating the repair of the intestinal barrier [87, 88].

ApoEVs have shown a significant potential for the modification of immunological responses, especially in treating autoimmune diseases. These EVs can be used as a treatment by aiding macrophages in producing transforming growth factor beta (TGFβ). They reduce inflammation and particularly alleviate gastrointestinal inflammation in mice with experimental colitis by lowering IFN-γ^+^ inflammatory T cells and increasing regulatory T cells (Tregs) in a TGFβ-dependent manner. Despite various challenges, such as allergic reactions, varied apoptotic conditions, and unpredictable cell death rates, the infusion of apoptotic cells is advantageous for treating autoimmune diseases, including graft-versus-host disease (GvHD) [89]. ApoEVs provide a promising option by substituting the therapeutic advantages of intact dead cells. When administered in a living organism, ApoEVs reduce inflammation, enhance the development of Tregs, and promote the production of TGFβ by macrophages. ApoEVs derived from mouse and human T cell lines have the same functional features, demonstrating a conserved ability to produce TGFβ [90]. Consistent with prior research indicating the role of FOXO3 as a crucial transcription factor for TGFβ in human monocytes, suppressing the *FOXO3* gene significantly reduces TGFβ production [91]. Utilizing ApoEV-based therapeutics for human autoimmune diseases presents a promising and potentially groundbreaking alternative.

**Fig. 6:**
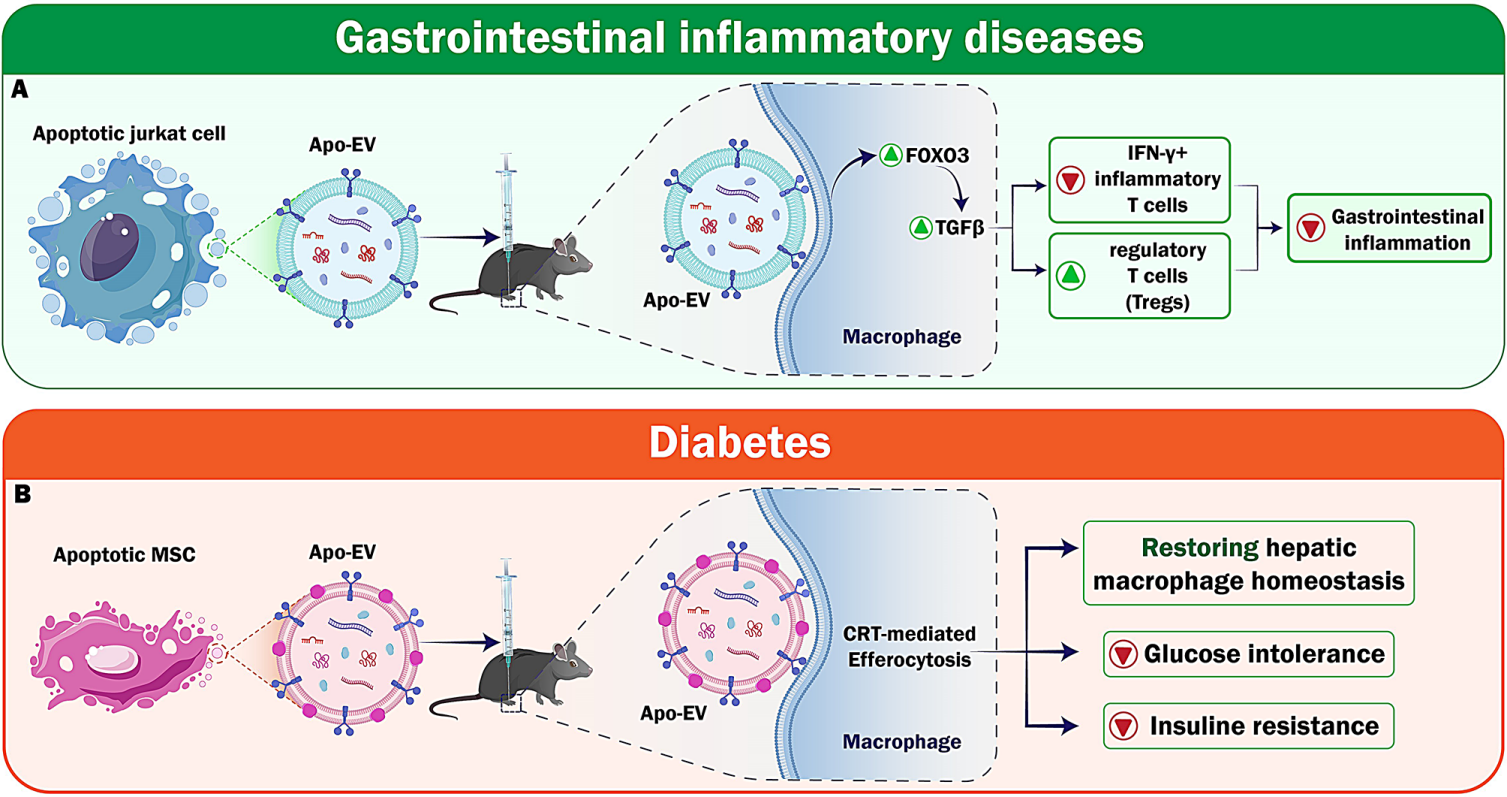
ApoEVs can be used to target gastrointestinal inflammatory disease and diabetes due to their anti-inflammatory properties. **A**) For gastrointestinal inflammatory disease, Apo EVs boost regulatory T cells and suppress inflammatory T cells via the FOXO3/TGF-β axis. **B**) In diabetes, ApoEVs promote CRT-mediated efferocytosis, reducing insulin resistance and glucose intolerance. CRT, Calreticulin

The creation of substitute anti-inflammatory therapies is essential to the resolution of inflammatory diseases. A possible dual-target strategy combines the specific ApoBDs produced by macrophages with the intrinsic chemotactic abilities of T cells to target inflamed regions. The selectivity of EVs further supports their feasibility for targeted drug administration. The development of chimeric apoptotic bodies (cABs) addressed the issue of inadequate therapeutic drug accumulation in inflammatory regions. cABs ensure enhanced accumulation at the site of inflammation via interactions with adhesion molecules on the surfaces of target cells and EVs. Particularly, cABs have shown little in vivo toxicity, underscoring their excellent safety and biocompatibility. Verifying the effectiveness of customized EVs in the management of inflammation, this innovative idea integrates vesicle membranes with diverse biological properties into modular delivery vehicles. While the focus on inflammatory macrophages provides valuable new insights, further investigation into the potential immune regulating effects of cABs on other immune cells is warranted. The potential for this approach to be translated into clinical practice may be enhanced by comparative studies between cABs and traditional treatment drugs, such as corticosteroids. Naturally occurring membrane-based engineered EVs represent a significant advancement in bioinspired EVs for targeted therapy. Standardization of manipulation, characterization, assessment procedures, consistent cell growth settings, and vesicle separation methods are also necessary for the successful clinical translation of these bioengineered EVs [92]. By highlighting the potential of cABs as a secure and effective method of treating inflammation, this tale paves the way for future developments in targeted drug delivery systems for inflammatory disorders. A new chapter in developing biocompatible medications that ensure safety and specificity in treating challenging inflammatory illnesses has been reached with natural membrane vesicles and modular delivery systems (Fig. 6).

### Diabetes

Diabetes is another immune system related disorder that poses severe challenges. The therapeutic potential of ApoEVs for patients with T2D has attracted attention for their power to restore hepatic macrophage homeostasis obtained from MSCs. Transcriptional reprogramming follows the efferocytosis of ApoEVs by macrophages, transforming them into anti-inflammatory agents and thereby reducing their liver accumulation. An essential component of this process is calreticulin (CRT), which presents on the surface of ApoEVs. As a crucial “eat-me” signal, CRT regulates macrophage activity and enables them to efferocytosis of ApoEVs. Studies have shown that restoring the homeostasis of hepatic macrophages through CRT-mediated efferocytosis of ApoEVs can help reduce significant T2D symptoms like glucose intolerance and insulin resistance (Fig. 6). These findings have advanced our understanding of the mechanisms and biological consequences of efferocytosis of ApoEVs. The exceptionally negative charge of ApoEVs most likely results in various phagocytic responses. Further studies are required to define the exact signaling molecules and pathways engaged in this process [93] Table 2.

**Table 2:** The implementation of apoptotic vesicles in inflammatory disease

Source of apoptotic vesicles	Conditions	Modifications	Mechanism	Reference
-MQ -Osteoclast	RA	--	-M2 MQ polarization -Chondrogenesis promotion -Osteoclast formation inhibition	[17]
M2MQ	OA	--	M2 polarization through miR-21-5p	[16]
ApoBD-inspired nanoliposomes containing phosphatidylserine	OA	Phosphatidylserine decoration of NPs JQ1-loaded polymer-stabilized liposomes (JQ1@PSLs)	BRD4 blocking-based M2 polarization	[86]
MSCs	OA	--	-Stopped Th17 differentiation -Inflammation and joint damage inhibition -Pro-inflammatory cytokines Reduction	[18]
T cells	OA	miR-124	-Induces M2 repolarization -Alleviates synovial inflammation	[94]
T cells	Radiation enteritis	--	-Inhibition of the cGAS-STING -Alleviation of radiation enteritis	[95]
-Thymocytes -Jurkat cells	Colitis	--	-Lowering IFN-γ^+^ inflammatory T cells and increasing Tregs in a TGFβ-dependent manner	[90]
MQ	IBD	MSNs were preloaded with anti-inflammatory agents (miR-21 or curcumin) and modified with stimuli-responsive molecules to achieve accurate cargo release at designated locations.	Induction of M2 polarization	[92]

MQ, Macrophage; RA, Rheumatoid Arthritis; OA, osteoarthritis; IBD, inflammatory bowel disease

### Remaining limitations

Several significant restrictions and challenges exist regarding the use of ApoEVs as anti-inflammatory treatments, which may lead to unforeseen immune system reactions. A substantial hurdle is the inherent variability of ApoEVs, which include various subtypes, including ApoBDs and apoptotic MVs. Standardizing the therapeutic application of these subtypes is difficult due to their notable differences in size, molecular makeup, and immunological properties. For example, while smaller ApoEVs may not trigger the same inflammatory responses, larger ones can enhance neutrophil movement, exacerbating inflammation. The absence of explicit criteria to distinguish between these subtypes complicates matters further and makes achieving consistent therapeutic outcomes across different applications more difficult [30].

Additionally, there is a risk of adverse effects stemming from the immunogenicity of ApoEVs. Although apoptosis is typically viewed as a process that promotes immune tolerance, specific apoptotic cells might inadvertently awaken immune responses, especially if they harbor pro-inflammatory cytokines or DAMPs. For instance, ApoEVs could contain bioactive elements like IL-1α or DNA fragments that can unintentionally activate immune cells, potentially leading to complications such as autoimmune reactions or inflammation. The dual nature of apoptosis, which can either provoke inflammation or encourage immune tolerance, underscores the complexities of using ApoEVs for therapeutic purposes.

Moreover, the existing challenges in accurately characterizing and isolating ApoEVs represent significant obstacles to their therapeutic application. The heterogeneous populations generated by current isolation techniques may not accurately correspond to the desired treatment subtype. As a result of this impure composition, findings from preclinical studies can vary, making it difficult to assess the safety and effectiveness of these vesicles. To fully leverage their potential as anti-inflammatory treatments while minimizing the risk of adverse immune responses, it is crucial to ensure that only well-defined and functionally characterized ApoEVs are utilized in therapeutic contexts [96].

### Future perspectives

The future of ApoEVs in advanced medicine is incredibly bright, filled with a wealth of opportunities ready to be explored. These innovative EVs have the potential to revolutionize vascular remodeling, effectively addressing the critical challenge of dysfunctional vascular structures that often lead to hypoxia, a significant contributor to tumor progression. In addition, ApoEVs have shown exceptional promise in stimulating hair growth, offering powerful alternatives to traditional therapies like minoxidil. This breakthrough opens the door to exciting new treatment strategies for managing alopecia.

Moreover, the unique characteristics of ApoEVs allow for targeted interventions in hypoxic areas of the body, unlocking transformative possibilities for in vivo gene therapy applications that remain mostly uncharted. We hypothesize by packaging therapeutic agents within ApoEVs, we could drive significant advancements for patients facing complex ocular conditions, such as retinal artery occlusion. Additionally, research suggests that ApoEVs could play a vital role in addressing diabetes, and their remarkable anti-inflammatory properties may also benefit a wide range of autoimmune diseases.

## Conclusion

ApoEVs are emerging as exciting tools in modern medicine, highlighting their potential across a variety of applications. From revolutionizing drug delivery to modulating immune responses and aiding in tissue repair, these tiny vesicles have much to offer. They tackle significant hurdles, including the complicated process of exosome extraction and the limitations of traditional inflammation, ischemia, and autoimmunity treatments.

What truly sets ApoEVs apart is their remarkable anti-inflammatory properties and regenerative capabilities. Delivering therapeutic cargo precisely where needed opens new possibilities for cancer treatment, helps manage ischemic conditions, and relieves autoimmune diseases. Additionally, their innate ability to target hypoxic areas enhances their adaptability, making them invaluable for targeted therapies and promoting vascular remodeling. With such promising features, ApoEVs could lead in a new era of more effective, tailored treatments in medicine. Nevertheless, the challenges associated with the accurate characterization and isolation of various subtypes of ApoEVs, along with their potential adverse effects resulting from immunogenicity, need careful consideration.

## Data Availability

No datasets were generated or analysed during the current study.

## References

[1] Singh R, Letai A, Sarosiek K. Regulation of apoptosis in health and disease: the balancing act of BCL-2 family proteins. Nat Rev Mol Cell Biol. 2019;20(3):175–93. 30655609 10.1038/s41580-018-0089-8 PMC7325303

[2] Boada-Romero E, Martinez J, Heckmann BL, Green DR. The clearance of dead cells by efferocytosis. Nat Rev Mol Cell Biol. 2020;21(7):398–414. 32251387 10.1038/s41580-020-0232-1 PMC7392086

[3] Doran AC, Yurdagul A, Tabas I. Efferocytosis in health and disease. Nat Rev Immunol. 2020;20(4):254–67. 31822793 10.1038/s41577-019-0240-6 PMC7667664

[4] Kumar MA, Baba SK, Sadida HQ, Marzooqi SAl, Jerobin J, Altemani FH, et al. Extracellular vesicles as tools and targets in therapy for diseases. Sig Transduct Target Ther. 2024;9(1):27. 10.1038/s41392-024-01735-1 PMC10838959 38311623

[5] He J, Ren W, Wang W, Han W, Jiang L, Zhang D, et al. Exosomal targeting and its potential clinical application. Drug Deliv Transl Res. 2022;12(10):2385–402. 34973131 10.1007/s13346-021-01087-1 PMC9458566

[6] Kakarla R, Hur J, Kim YJ, Kim J, Chwae YJ. Apoptotic cell-derived exosomes: messages from dying cells. Exp Mol Med. 2020;52(1):1–6. 31915368 10.1038/s12276-019-0362-8 PMC7000698

[7] Phan TK, Ozkocak DC, Poon IKH. Unleashing the therapeutic potential of apoptotic bodies. Biochem Soc Trans. 2020;48(5):2079–88. 32869835 10.1042/BST20200225 PMC7609033

[8] Singh V, Khurana A, Navik U, Allawadhi P, Bharani KK, Weiskirchen R. Apoptosis and Pharmacological therapies for targeting thereof for Cancer therapeutics. Sci. 2022;4(2):15.

[9] Wang J, Cao Z, Wang P, Zhang X, Tang J, He Y, et al. Apoptotic extracellular vesicles ameliorate multiple myeloma by restoring Fas-Mediated apoptosis. ACS Nano. 2021;15(9):14360–72. 34506129 10.1021/acsnano.1c03517

[10] Horrevorts S, Ven, Hulst, Hof D, et al. Glycan-Modified Melanoma-Derived apoptotic extracellular vesicles as antigen source for Anti-Tumor vaccination. Cancers. 2019;11(9):1266. 31466401 10.3390/cancers11091266 PMC6769957

[11] Wang P, Cao J, Feng Z, Tang Y, Han X, Mao T, et al. Oroxylin a promoted apoptotic extracellular vesicles transfer of glycolytic kinases to remodel immune microenvironment in hepatocellular carcinoma model. Eur J Pharmacol. 2023;957:176037. 37660969 10.1016/j.ejphar.2023.176037

[12] Jing Y, Zhao W, Zhou Z, Wang W, Niu Y, He X et al. Apoptotic vesicles modulate endothelial metabolism and ameliorate ischemic retinopathy via PD1/PDL1 Axis. Adv Healthc Mater. 2024;2303527. 10.1002/adhm.202303527 PMC11468456 38411334

[13] Lei F, Huang Z, Ou Q, Li J, Liu M, Ma L, et al. Apoptotic vesicles rejuvenate mesenchymal stem cells via Rab7-mediated autolysosome formation and alleviate bone loss in aging mice. Nano Res. 2023;16(1):822–33.

[14] Mehrotra P, Ravichandran KS. Drugging the efferocytosis process: concepts and opportunities. Nat Rev Drug Discov. 2022;21(8):601–20. 35650427 10.1038/s41573-022-00470-y PMC9157040

[15] Brandel V, Schimek V, Göber S, Hammond T, Brunnthaler L, Schrottmaier WC, et al. Hepatectomy-induced apoptotic extracellular vesicles stimulate neutrophils to secrete regenerative growth factors. J Hepatol. 2022;77(6):1619–30. 35985549 10.1016/j.jhep.2022.07.027

[16] Qin L, Yang J, Su X, Xilan Li, Lei Y, Dong L, et al. The miR-21-5p enriched in the apoptotic bodies of M2 macrophage-derived extracellular vesicles alleviates osteoarthritis by changing macrophage phenotype. Genes Dis. 2023;10(3):1114–29. 37396516 10.1016/j.gendis.2022.09.010 PMC10308169

[17] Li X, Li S, Fu X, Wang Y. Apoptotic extracellular vesicles restore homeostasis of the articular microenvironment for the treatment of rheumatoid arthritis. Bioactive Mater. 2024;35:564–76. 10.1016/j.bioactmat.2023.11.019 PMC10925912 38469201

[18] Wang R, Hao M, Kou X, Sui B, Sanmillan ML, Zhang X, et al. Apoptotic vesicles ameliorate lupus and arthritis via phosphatidylserine-mediated modulation of T cell receptor signaling. Bioactive Mater. 2023;25:472–84. 10.1016/j.bioactmat.2022.07.026 PMC10087106 37056273

[19] Elmore S, Apoptosis. A review of programmed cell death. Toxicol Pathol. 2007;35(4):495–516. 17562483 10.1080/01926230701320337 PMC2117903

[20] Wani AK, Akhtar N, Mir TUG, Singh R, Jha PK, Mallik SK, et al. Targeting apoptotic pathway of Cancer cells with phytochemicals and Plant-Based nanomaterials. Biomolecules. 2023;13(2):194. 36830564 10.3390/biom13020194 PMC9953589

[21] Van Opdenbosch N, Lamkanfi M. Caspases in cell death, inflammation, and disease. Immunity. 2019;50(6):1352–64. 31216460 10.1016/j.immuni.2019.05.020 PMC6611727

[22] Essola JM, Zhang M, Yang H, Li F, Xia B, Mavoungou JF, et al. Exosome regulation of immune response mechanism: pros and cons in immunotherapy. Bioactive Mater. 2024;32:124–46. 10.1016/j.bioactmat.2023.09.018 PMC10622742 37927901

[23] Wen J, Creaven D, Luan X, Wang J. Comparison of immunotherapy mediated by apoptotic bodies, microvesicles and exosomes: apoptotic bodies’ unique anti-inflammatory potential. J Transl Med. 2023;21(1):478. 37461033 10.1186/s12967-023-04342-w PMC10353199

[24] Gregory CD, Rimmer MP. Extracellular vesicles arising from apoptosis: forms, functions, and applications. J Pathol. 2023;260(5):592–608. 37294158 10.1002/path.6138 PMC10952477

[25] Li X, Liu Y, Liu X, Du J, Bhawal UK, Xu J, et al. Advances in the therapeutic effects of apoptotic bodies on systemic diseases. IJMS. 2022;23(15):8202. 35897778 10.3390/ijms23158202 PMC9331698

[26] Tual-Chalot S, Leonetti D, Andriantsitohaina R, Martinez MC. Microvesicles: intercellular vectors of biological messages. Mol Interv. 2011;11(2):88–94. 21540467 10.1124/mi.11.2.5

[27] Jianbin L, Yiping H, Yueqin Z, Pengcheng L, Mengxia L, Min Z et al. S1P/S1PR signaling pathway advancements in autoimmune diseases. Biomol Biomed [Internet]. 2023 Jul 27 Available from: https://www.bjbms.org/ojs/index.php/bjbms/article/view/9082 10.17305/bb.2023.9082 PMC10655875 37504219

[28] Park SJ, Kim JM, Kim J, Hur J, Park S, Kim K et al. Molecular mechanisms of biogenesis of apoptotic exosome-like vesicles and their roles as damage-associated molecular patterns. Proc Natl Acad Sci USA [Internet]. 2018;115(50). Available from: 10.1073/pnas.1811432115 10.1073/pnas.1811432115 PMC6294905 30463946

[29] Riwaldt S, Corydon TJ, Pantalone D, Sahana J, Wise P, Wehland M, et al. Role of apoptosis in wound healing and apoptosis alterations in microgravity. Front Bioeng Biotechnol. 2021;9:679650. 34222218 10.3389/fbioe.2021.679650 PMC8248797

[30] Zou X, Lei Q, Luo X, Yin J, Chen S, Hao C, et al. Advances in biological functions and applications of apoptotic vesicles. Cell Commun Signal. 2023;21(1):260. 37749626 10.1186/s12964-023-01251-9 PMC10519056

[31] Mohammad RM, Muqbil I, Lowe L, Yedjou C, Hsu HY, Lin LT, et al. Broad targeting of resistance to apoptosis in cancer. Sem Cancer Biol. 2015;35:S78–103. 10.1016/j.semcancer.2015.03.001 PMC4720504 25936818

[32] Liu YT, Sun ZJ. Turning cold tumors into hot tumors by improving T-cell infiltration. Theranostics. 2021;11(11):5365–86. 33859752 10.7150/thno.58390 PMC8039952

[33] Wu TD, Madireddi S, De Almeida PE, Banchereau R, Chen YJJ, Chitre AS, et al. Peripheral T cell expansion predicts tumour infiltration and clinical response. Nature. 2020;579(7798):274–8. 32103181 10.1038/s41586-020-2056-8

[34] Decout A, Katz JD, Venkatraman S, Ablasser A. The cGAS–STING pathway as a therapeutic target in inflammatory diseases. Nat Rev Immunol. 2021;21(9):548–69. 33833439 10.1038/s41577-021-00524-z PMC8029610

[35] Garland KM, Sheehy TL, Wilson JT. Chemical and biomolecular strategies for STING pathway activation in Cancer immunotherapy. Chem Rev. 2022;122(6):5977–6039. 35107989 10.1021/acs.chemrev.1c00750 PMC8994686

[36] Bao P, Zheng ZT, Ye JJ, Zhang XZ. Apoptotic Body-Mediated intracellular delivery strategy for enhanced STING activation and improved tumor immunogenicity. Nano Lett. 2022;22(6):2217–27. 35254071 10.1021/acs.nanolett.1c03996

[37] Tiwari A, Trivedi R, Lin SY. Tumor microenvironment: barrier or opportunity towards effective cancer therapy. J Biomed Sci. 2022;29(1):83. 36253762 10.1186/s12929-022-00866-3 PMC9575280

[38] Li T, Jiao J, Ke H, Ouyang W, Wang L, Pan J, et al. Role of exosomes in the development of the immune microenvironment in hepatocellular carcinoma. Front Immunol. 2023;14:1200201. 37457718 10.3389/fimmu.2023.1200201 PMC10339802

[39] Zhao Z, Ukidve A, Kim J, Mitragotri S. Targeting strategies for Tissue-Specific drug delivery. Cell. 2020;181(1):151–67. 32243788 10.1016/j.cell.2020.02.001

[40] Sheng S, Yu X, Xing G, Jin L, Zhang Y, Zhu D, et al. An apoptotic Body-based vehicle with navigation for Photothermal‐Immunotherapy by precise delivery and tumor microenvironment regulation. Adv Funct Mater. 2023;33(5):2212118.

[41] Cao Z, Li P, Li Y, Zhang M, Hao M, Li W, et al. Encapsulation of Nano-Bortezomib in apoptotic stem Cell‐Derived vesicles for the treatment of multiple myeloma. Small. 2023;19(40):2301748. 10.1002/smll.202301748 37282762

[42] Gonzalez-Avila G, Sommer B, Mendoza-Posada DA, Ramos C, Garcia-Hernandez AA, Falfan-Valencia R. Matrix metalloproteinases participation in the metastatic process and their diagnostic and therapeutic applications in cancer. Crit Rev Oncol/Hematol. 2019;137:57–83. 31014516 10.1016/j.critrevonc.2019.02.010

[43] Shlomovitz I, Speir M, Gerlic M. Flipping the dogma – phosphatidylserine in non-apoptotic cell death. Cell Commun Signal. 2019;17(1):139. 31665027 10.1186/s12964-019-0437-0 PMC6819419

[44] Liu Y, Wang J, Zhang J, Marbach S, Xu W, Zhu L. Targeting Tumor-Associated macrophages by MMP2-Sensitive apoptotic Body-Mimicking nanoparticles. ACS Appl Mater Interfaces. 2020;12(47):52402–14. 33169982 10.1021/acsami.0c15983 PMC8229024

[45] Navik U, Rawat PS, Allawadhi P, Khurana A, Banothu AK, Bharani KK. Evolution of Zebrafish as a Novel Pharmacological Model in Endocrine Research. In: Bhandari PR, Bharani KK, Khurana A, editors. Zebrafish Model for Biomedical Research [Internet]. Singapore: Springer Nature Singapore; 2022. pp. 93–141. Available from: https://link.springer.com/10.1007/978-981-16-5217-2_6

[46] Haque E, Ward AC. Zebrafish as a model to evaluate nanoparticle toxicity. Nanomaterials. 2018;8(7):561. 30041434 10.3390/nano8070561 PMC6071110

[47] Gadige A, Gunturu NT, Khurana A, Allawadhi P, Khurana I, Banothu AK et al. Zebrafish as a Novel Pharmacological Screening Model for Drug Discovery and Development Against Hematological Disorders. In: Bhandari PR, Bharani KK, Khurana A, editors. Zebrafish Model for Biomedical Research [Internet]. Singapore: Springer Nature Singapore; 2022. pp. 259–87. Available from: https://link.springer.com/10.1007/978-981-16-5217-2_12

[48] Jin L, Sheng S, Zhang Y, Sun W, Mei L, Zhu D, et al. An optically responsive cancer vaccine for inducing robust anti-tumor immunity by apoptotic body carrying nanoadjuvants. Chem Eng J. 2024;496:153721.

[49] Sachdeva P, Kaur K, Fatima S, Mahak F, Noman M, Siddenthi SM et al. Advancements in Myocardial Infarction Management: Exploring Novel Approaches and Strategies. Cureus [Internet]. 2023; Available from: https://www.cureus.com/articles/188806-advancements-in-myocardial-infarction-management-exploring-novel-approaches-and-strategies 10.7759/cureus.45578 PMC10587445 37868550

[50] Thomas TP, Grisanti LA. The dynamic interplay between cardiac inflammation and fibrosis. Front Physiol. 2020;11:529075. 33041853 10.3389/fphys.2020.529075 PMC7522448

[51] Lee J, Sim W, Park H, Park B, Joung YK. Targeted delivery of apoptotic Cell-Derived nanovesicles prevents cardiac remodeling and attenuates cardiac function exacerbation. Adv Funct Mater. 2023;33(23):2210864.

[52] Dang Q, Sun Z, Wang Y, Wang L, Liu Z, Han X. Ferroptosis: a double-edged sword mediating immune tolerance of cancer. Cell Death Dis. 2022;13(11):925. 36335094 10.1038/s41419-022-05384-6 PMC9637147

[53] Jiang X, Stockwell BR, Conrad M. Ferroptosis: mechanisms, biology and role in disease. Nat Rev Mol Cell Biol. 2021;22(4):266–82. 33495651 10.1038/s41580-020-00324-8 PMC8142022

[54] Wen Q, Liu J, Kang R, Zhou B, Tang D. The release and activity of HMGB1 in ferroptosis. Biochem Biophys Res Commun. 2019;510(2):278–83. 30686534 10.1016/j.bbrc.2019.01.090

[55] Yu C, Xiao JH. The Keap1-Nrf2 system: A mediator between oxidative stress and aging. Rupasinghe HPV. Editor Oxidative Med Cell Longev. 2021;2021:1–16. 10.1155/2021/6635460 PMC8106771 34012501

[56] Liu L, Ye Y, Lin R, Liu T, Wang S, Feng Z, et al. Ferroptosis: a promising candidate for exosome-mediated regulation in different diseases. Cell Commun Signal. 2024;22(1):6. 38166927 10.1186/s12964-023-01369-w PMC11057189

[57] Liu D, Kou X, Chen C, Liu S, Liu Y, Yu W, et al. Circulating apoptotic bodies maintain mesenchymal stem cell homeostasis and ameliorate osteopenia via transferring multiple cellular factors. Cell Res. 2018;28(9):918–33. 30030518 10.1038/s41422-018-0070-2 PMC6123409

[58] Yu G, Chen Y, Yang N, Zhang H, Zhang X, Geng Y et al. Apoptotic bodies derived from Fibroblast-Like cells in subcutaneous connective tissue inhibit ferroptosis in ischaemic flaps via the miR‐339‐5p/KEAP1/Nrf2 Axis. Adv Sci. 2024;2307238. 10.1002/advs.202307238 PMC11200024 38639443

[59] Ganguly P, El-Jawhari JJ, Giannoudis PV, Burska AN, Ponchel F, Jones EA. Age-related changes in bone marrow mesenchymal stromal cells: A potential impact on osteoporosis and osteoarthritis development. Cell Transpl. 2017;26(9):1520–9. 10.1177/0963689717721201 PMC5680949 29113463

[60] Volkova MV, Shen N, Polyanskaya A, Qi X, Boyarintsev VV, Kovaleva EV, et al. Tissue-Oxygen-Adaptation of bone Marrow-Derived mesenchymal stromal cells enhances their Immunomodulatory and Pro-Angiogenic capacity, resulting in accelerated healing of chemical burns. IJMS. 2023;24(4):4102. 36835513 10.3390/ijms24044102 PMC9963537

[61] Pulido-Escribano V, Torrecillas-Baena B, Camacho-Cardenosa M, Dorado G, Gálvez-Moreno MÁ, Casado-Díaz A. Role of hypoxia preconditioning in therapeutic potential of mesenchymal stem-cell-derived extracellular vesicles. WJSC. 2022;14(7):453–72. 36157530 10.4252/wjsc.v14.i7.453 PMC9350626

[62] Tan F, Li X, Wang Z, Li J, Shahzad K, Zheng J. Clinical applications of stem cell-derived exosomes. Sig Transduct Target Ther. 2024;9(1):17. 10.1038/s41392-023-01704-0 PMC10784577 38212307

[63] Ding Z, Yan Z, Yuan X, Tian G, Wu J, Fu L, et al. Apoptotic extracellular vesicles derived from hypoxia-preconditioned mesenchymal stem cells within a modified gelatine hydrogel promote osteochondral regeneration by enhancing stem cell activity and regulating immunity. J Nanobiotechnol. 2024;22(1):74. 10.1186/s12951-024-02333-7 PMC10885680 38395929

[64] Leung KS, Shirazi S, Cooper LF, Ravindran S. Biomaterials and extracellular vesicle delivery: current status, applications and challenges. Cells. 2022;11(18):2851. 36139426 10.3390/cells11182851 PMC9497093

[65] Zhou J, Li Q, Tian Z, Yao Q, Zhang M. Recent advances in 3D bioprinted cartilage-mimicking constructs for applications in tissue engineering. Mater Today Bio. 2023;23:100870. 38179226 10.1016/j.mtbio.2023.100870 PMC10765242

[66] Mutharasan RK, Nagpal V, Ichikawa Y, Ardehali H. microRNA-210 is upregulated in hypoxic cardiomyocytes through Akt- and p53-dependent pathways and exerts cytoprotective effects. Am J Physiol Heart Circ Physiol. 2011;301(4):H1519–30. 21841015 10.1152/ajpheart.01080.2010 PMC3197368

[67] Yu L, Zhu G, Zhang Z, Yu Y, Zeng L, Xu Z, et al. Apoptotic bodies: bioactive treasure left behind by the dying cells with robust diagnostic and therapeutic application potentials. J Nanobiotechnol. 2023;21(1):218. 10.1186/s12951-023-01969-1 PMC10337089 37434199

[68] Ling Z, Guo S, Xie H, Chen X, Yu K, Jiang H, et al. Synergistic effects of cerium-containing bioactive glasses and apoptotic extracellular vesicles alleviate bisphosphonate-related osteonecrosis of jaw. Appl Mater Today. 2024;38:102177.

[69] Zhu Y, Zhang X, Yang K, Shao Y, Gu R, Liu X, et al. Macrophage-derived apoptotic vesicles regulate fate commitment of mesenchymal stem cells via miR155. Stem Cell Res Ther. 2022;13(1):323. 35842708 10.1186/s13287-022-03004-w PMC9288680

[70] Brodeur A, Migneault F, Lanoie M, Beillevaire D, Turgeon J, Karakeussian-Rimbaud A, et al. Apoptotic exosome-like vesicles transfer specific and functional mRNAs to endothelial cells by phosphatidylserine-dependent macropinocytosis. Cell Death Dis. 2023;14(7):449. 37474514 10.1038/s41419-023-05991-x PMC10359336

[71] Yu P, Deng S, Yuan X, Pan J, Xu J. Extracellular Vesicles and Vascular Inflammation. In: Xiao J, editor. Extracellular Vesicles in Cardiovascular and Metabolic Diseases [Internet]. Singapore: Springer Nature Singapore; 2023 [cited 2024 May 31]. pp. 105–17. Available from: https://link.springer.com/10.1007/978-981-99-1443-2_7

[72] Migneault F, Dieudé M, Turgeon J, Beillevaire D, Hardy MP, Brodeur A, et al. Apoptotic exosome-like vesicles regulate endothelial gene expression, inflammatory signaling, and function through the NF-κB signaling pathway. Sci Rep. 2020;10(1):12562. 32724121 10.1038/s41598-020-69548-0 PMC7387353

[73] Raziyeva K, Kim Y, Zharkinbekov Z, Kassymbek K, Jimi S, Saparov A. Immunology of acute and chronic wound healing. Biomolecules. 2021;11(5):700. 34066746 10.3390/biom11050700 PMC8150999

[74] Shen S, Shao Y, Li C. Different types of cell death and their shift in shaping disease. Cell Death Discov. 2023;9(1):284. 37542066 10.1038/s41420-023-01581-0 PMC10403589

[75] Wang Y, Jing L, Lei X, Ma Z, Li B, Shi Y, et al. Umbilical cord mesenchymal stem cell-derived apoptotic extracellular vesicles ameliorate cutaneous wound healing in type 2 diabetic mice via macrophage pyroptosis Inhibition. Stem Cell Res Ther. 2023;14(1):257. 37726853 10.1186/s13287-023-03490-6 PMC10510296

[76] Qu Y, He Y, Meng B, Zhang X, Ding J, Kou X, et al. Apoptotic vesicles inherit SOX2 from pluripotent stem cells to accelerate wound healing by energizing mesenchymal stem cells. Acta Biomater. 2022;149:258–72. 35830925 10.1016/j.actbio.2022.07.009

[77] Ma L, Chen C, Liu D, Huang Z, Li J, Liu H, et al. Apoptotic extracellular vesicles are metabolized regulators nurturing the skin and hair. Bioactive Mater. 2023;19:626–41. 10.1016/j.bioactmat.2022.04.022 PMC9109130 35600968

[78] Rai R, Regan L. Recurrent miscarriage. Lancet. 2006;368(9535):601–11. 16905025 10.1016/S0140-6736(06)69204-0

[79] Xin L, Wei C, Tong X, Dai Y, Huang D, Chen J, et al. In situ delivery of apoptotic bodies derived from mesenchymal stem cells via a hyaluronic acid hydrogel: A therapy for intrauterine adhesions. Bioactive Mater. 2022;12:107–19. 10.1016/j.bioactmat.2021.10.025 PMC8777284 35087967

[80] Black RJ, Cross M, Haile LM, Culbreth GT, Steinmetz JD, Hagins H, et al. Global, regional, and National burden of rheumatoid arthritis, 1990–2020, and projections to 2050: a systematic analysis of the global burden of disease study 2021. Lancet Rheumatol. 2023;5(10):e594–610. 37795020 10.1016/S2665-9913(23)00211-4 PMC10546867

[81] Bullock J, Rizvi SAA, Saleh AM, Ahmed SS, Do DP, Ansari RA, et al. Rheumatoid arthritis: A brief overview of the treatment. Med Princ Pract. 2018;27(6):501–7. 30173215 10.1159/000493390 PMC6422329

[82] Fernandes TL, Gomoll AH, Lattermann C, Hernandez AJ, Bueno DF, Amano MT. Macrophage: A potential target on cartilage regeneration. Front Immunol. 2020;11:111. 32117263 10.3389/fimmu.2020.00111 PMC7026000

[83] Zhang G, Xue H, Sun D, Yang S, Tu M, Zeng R. Soft apoptotic-cell-inspired nanoparticles persistently bind to macrophage membranes and promote anti-inflammatory and pro-healing effects. Acta Biomater. 2021;131:452–63. 34245890 10.1016/j.actbio.2021.07.002

[84] Wang N, Wu R, Tang D, Kang R. The BET family in immunity and disease. Sig Transduct Target Ther. 2021;6(1):23. 10.1038/s41392-020-00384-4 PMC7813845 33462181

[85] Huang Z, Yang R, Zhang L, Zhu M, Zhang C, Wen J, et al. BRD4 Inhibition alleviates mechanical stress-induced TMJ OA-like pathological changes and attenuates TREM1-mediated inflammatory response. Clin Epigenet. 2021;13(1):10. 10.1186/s13148-021-01008-6 PMC7809762 33446277

[86] Xu YD, Liang XC, Li ZP, Wu ZS, Yang J, Jia SZ, et al. Apoptotic body-inspired nanotherapeutics efficiently attenuate osteoarthritis by targeting BRD4-regulated synovial macrophage polarization. Biomaterials. 2024;306:122483. 38330742 10.1016/j.biomaterials.2024.122483

[87] Moreira Lopes TC, Mosser DM, Gonçalves R. Macrophage polarization in intestinal inflammation and gut homeostasis. Inflamm Res. 2020;69(12):1163–72. 32886145 10.1007/s00011-020-01398-y

[88] Allawadhi P, Beyer G, Mahajan UM, Mayerle J. Novel insights into macrophage diversity during the course of pancreatitis. Gastroenterology. 2021;161(6):1802–5. 34587487 10.1053/j.gastro.2021.09.049

[89] Kleinclauss F, Perruche S, Masson E, De Carvalho Bittencourt M, Biichle S, Remy-Martin JP, et al. Intravenous apoptotic spleen cell infusion induces a TGF-β-dependent regulatory T-cell expansion. Cell Death Differ. 2006;13(1):41–52. 15962005 10.1038/sj.cdd.4401699 PMC3448559

[90] Chen H, Kasagi S, Chia C, Zhang D, Tu E, Wu R, et al. Extracellular vesicles from apoptotic cells promote TGFβ production in macrophages and suppress experimental colitis. Sci Rep. 2019;9(1):5875. 30971739 10.1038/s41598-019-42063-7 PMC6458171

[91] Lee JC, Espéli M, Anderson CA, Linterman MA, Pocock JM, Williams NJ, et al. Human SNP links differential outcomes in inflammatory and infectious disease to a FOXO3-Regulated pathway. Cell. 2013;155(1):57–69. 24035192 10.1016/j.cell.2013.08.034 PMC3790457

[92] Dou G, Tian R, Liu X, Yuan P, Ye Q, Liu J, et al. Chimeric apoptotic bodies functionalized with natural membrane and modular delivery system for inflammation modulation. Sci Adv. 2020;6(30):eaba2987. 32832662 10.1126/sciadv.aba2987 PMC7439513

[93] Zheng C, Sui B, Zhang X, Hu J, Chen J, Liu J, et al. Apoptotic vesicles restore liver macrophage homeostasis to counteract type 2 diabetes. J Extracell Vesicle. 2021;10(7):e12109. 10.1002/jev2.12109 PMC8144839 34084287

[94] Li Z, Cheng Q, Lin L, Fu X, Wang Y. Plasma Membrane-Derived biomimetic apoptotic nanovesicles targeting inflammation and cartilage degeneration for osteoarthritis. Small Methods. 2024;2400660. 10.1002/smtd.202400660 39036830

[95] Zhou Y, Bao L, Gong S, Dou G, Li Z, Wang Z, et al. T Cell-Derived apoptotic extracellular vesicles hydrolyze cGAMP to alleviate radiation enteritis via surface enzyme ENPP1. Adv Sci. 2024;11(31):2401634. 10.1002/advs.202401634 PMC11336903 38888507

[96] Miao X, Wu X, You W, He K, Chen C, Pathak JL, et al. Tailoring of apoptotic bodies for diagnostic and therapeutic applications:advances, challenges, and prospects. J Transl Med. 2024;22(1):810. 39218900 10.1186/s12967-024-05451-w PMC11367938

